# 20 years of herpes simplex virus type 2 (HSV-2) research in low-income and middle-income countries: systematic evaluation of progress made in addressing WHO priorities for research in HSV-2/HIV interactions, HSV-2 control and mathematical modelling

**DOI:** 10.1136/bmjgh-2024-015167

**Published:** 2024-07-04

**Authors:** Ela Mair Owen, Muna Jama, Belinder Nahal, Emily Clarke, Angela Obasi

**Affiliations:** 1 Liverpool School of Tropical Medicine, Liverpool, UK; 2 University of Liverpool, Liverpool, UK; 3 International Rescue Committee, Mogadishu, Somalia; 4 London School of Hygiene & Tropical Medicine, London, UK; 5 Axess Sexual Health, Liverpool University Hospitals NHS Foundation Trust, Liverpool, UK

**Keywords:** Global Health, Public Health, HIV, Review, Mathematical modelling

## Abstract

**Introduction:**

Reviewing and updating research priorities is essential to assess progress and to ensure optimal allocation of financial and human resources in research. In 2001, WHO held a research priority setting workshop for herpes simplex virus type 2 (HSV-2) research in low-income and middle-income countries (LMICs). This study aimed to describe progress between 2000 and 2020 in three of the five key research priority areas outlined in the workshop: HSV-2/HIV interactions, HSV-2 control measures and HSV-2 mathematical modelling. The remaining priorities are addressed in a companion paper.

**Method:**

A systematic literature search of MEDLINE, CINAHL, Global Health and Cochrane databases was carried out. Relevant primary research studies based in LMICs, written in English and published on 2000–2020 were included. Papers were screened by two independent reviewers, and suitable variables were selected for manual extraction from study texts. Data were organised into an Excel spreadsheet and analysed using IBM SPSS.

**Results:**

In total, 3214 discrete papers were identified, of which 180 were eligible for inclusion (HSV-2/HIV interactions, 98; control measures, 58; mathematical modelling, 24). Most studies were conducted in East Africa. The majority of the 2001 WHO HSV-2 research priorities were addressed at least in part. Overall, despite several studies describing a strong relationship between HSV-2 and the acquisition and transmission of HIV, HSV-2 control repeatedly demonstrated little effect on HIV shedding or transmission. Further, although mathematical modelling predicted that vaccines could significantly impact HSV-2 indicators, HSV-2 vaccine studies were few. Studies of antiviral resistance were also few.

**Conclusion:**

Since 2000, LMIC HSV-2 research addressing its control, HIV interactions and mathematical modelling has largely addressed the priorities set in the 2001 WHO HSV-2 workshop. However, key knowledge gaps remain in vaccine research, antiviral cost-effectiveness, antiviral resistance and specific geographical areas.

**WHAT IS ALREADY KNOWN ON THIS TOPIC**

- Herpes simplex virus type 2 (HSV-2) has a high disease burden in low-income and middle-income countries (LMICs) and is closely related to HIV. In 2001, WHO set some research priorities for HSV-2 in LMICs with the help of global experts. This study reviews the progress towards meeting these priorities.

**WHAT THIS STUDY ADDS**

- Although many of the priorities were addressed, knowledge gaps were identified within vaccine research, antiviral cost-effectiveness, antiviral resistance and specific geographical areas.

**HOW THIS STUDY MIGHT AFFECT RESEARCH, PRACTICE OR POLICY**

- This study identified unaddressed HSV-2 research priorities that may be utilised to formulate updated research priorities for HSV-2 in LMICs.

## Introduction

Herpes simplex virus type 2 (HSV-2) is a sexually transmitted infection which causes painful genital ulcers and affects over 400 million adults globally.^1^ Low-income and middle-income countries (LMICs) bear the greatest global burden of HSV-2 infection; for example, the population prevalence in Africa is over 30%, whereas in Europe, it is below 10%.^1^ Across all settings, women, men who have sex with men (MSM) and commercial sex workers are disproportionately affected.^2 3^ Cumulative evidence has implicated HSV-2 in HIV epidemics as a facilitator or HIV acquisition and transmission.^4 5^ Several studies have shown that people who are HSV-2 seropositive are up to five times more likely to acquire HIV, and people coinfected with HSV-2 and HIV are significantly more likely to transmit HIV to their sexual partners.^6–9^ Examination of this relationship and its potential as a means of HIV control have been major drivers of HSV-2 research in LMICs.

Against this background, in 2001, WHO held a 3-day workshop to identify priorities for research to address the high burden of HSV-2 in low-income and middle-income countries (LMICs).^10^ Delegates included 40 HSV-2 experts from 26 institutions in 12 countries further described in a companion paper.^11^ Research priorities were identified in five key areas: (1) the epidemiology of HSV-2, (2) the interaction between HSV-2 and HIV, (3) effective strategies for HSV-2 control, (4) mathematical modelling of HSV-2 and (5) HSV-2 diagnostics. Areas for further examination were explored in each area. These included evaluating and modelling the effectiveness of HSV-2 suppressive therapy and vaccination, the impact of antiherpetic therapy on HIV indicators and aciclovir resistance. Additionally, emphasis was put on the need to lobby for further vaccine development and affordable provision of aciclovir for low-income and middle-income countries.^12^

In order to achieve maximum benefit from priority setting activities, progress towards the defined priorities should be evaluated at appropriate time intervals.^13 14^ Further, since research priorities can influence the allocation of research funding, reviewing priorities allows optimal allocation of finite resources to maximise the likelihood of patient and population benefit.^14^ To our knowledge, there has been no published review of the progress towards the research priorities defined in 2001.

The current study therefore aimed to review the progress between 2000 and 2020 in addressing the 2001 WHO HSV-2 research priorities for LMICs in three out of the five key research priority areas identified by the workshop: HSV-2/HIV interactions (priority area 1), HSV-2 control measures (priority area 2) and HSV-2 mathematical modelling (priority area 3). Progress in the remaining priority areas is addressed in a companion paper.^11^

## Methods

A systematic scoping of the literature was performed to assess the research progress made in the HSV-2 control measures, interactions with HIV and mathematical modelling between 2000 and 2020. Data were collected on numbers of studies, their objectives, main findings and geographical locations. Data were also collected on sample size, study design, use of randomisation and sources of bias to allow the assessment of quality of the evidence in the articles.

The databases MEDLINE Complete, CINAHL, Global Health and The Cochrane Library were systematically searched for relevant literature using keywords and subject headings. Search terms were refined through preliminary scoping. Independent searches were performed for each priority area evaluated in this study. Search terms were chosen for each priority area, for ‘low and middle-income countries’ and for ‘HSV-2’ and combined using Boolean operators ‘OR’ and ‘AND’. For example, to address priority area 1, the keyword search strategy was as follows:

- (‘HSV-2’ OR ‘genital herpes’ OR ‘herpes genitalis’ OR ‘herpes simplex 2’ OR ‘human simplex virus 2’ OR ‘herpes virus 2’) **AND**
- (‘developing countr*’ OR ‘LMIC’ OR ‘low-to-middle income’ OR ‘low to middle income’ OR ‘low income’ OR ‘middle income’ OR ‘least developed countr*’ OR ‘less developed countr*’ OR ‘underdeveloped countr*’ OR ‘under developed nation*’ OR ‘poor countr*’ OR ‘third world countr*’ OR ‘third world nation*’ OR ‘least developed nation*’ OR ‘less developed nation*’ OR ‘global South’ OR ‘sub-Saharan Africa’ OR ‘Asia’ OR ‘South America’) **AND**
- (‘HIV’ OR ‘human immunodeficiency virus’ OR ‘AIDS’ OR ‘acquired immunodeficiency syndrome’)

See online supplemental appendix 1 for a comprehensive description of the search strategies.

10.1136/bmjgh-2024-015167.supp1 Supplementary data

**Figure 1:**
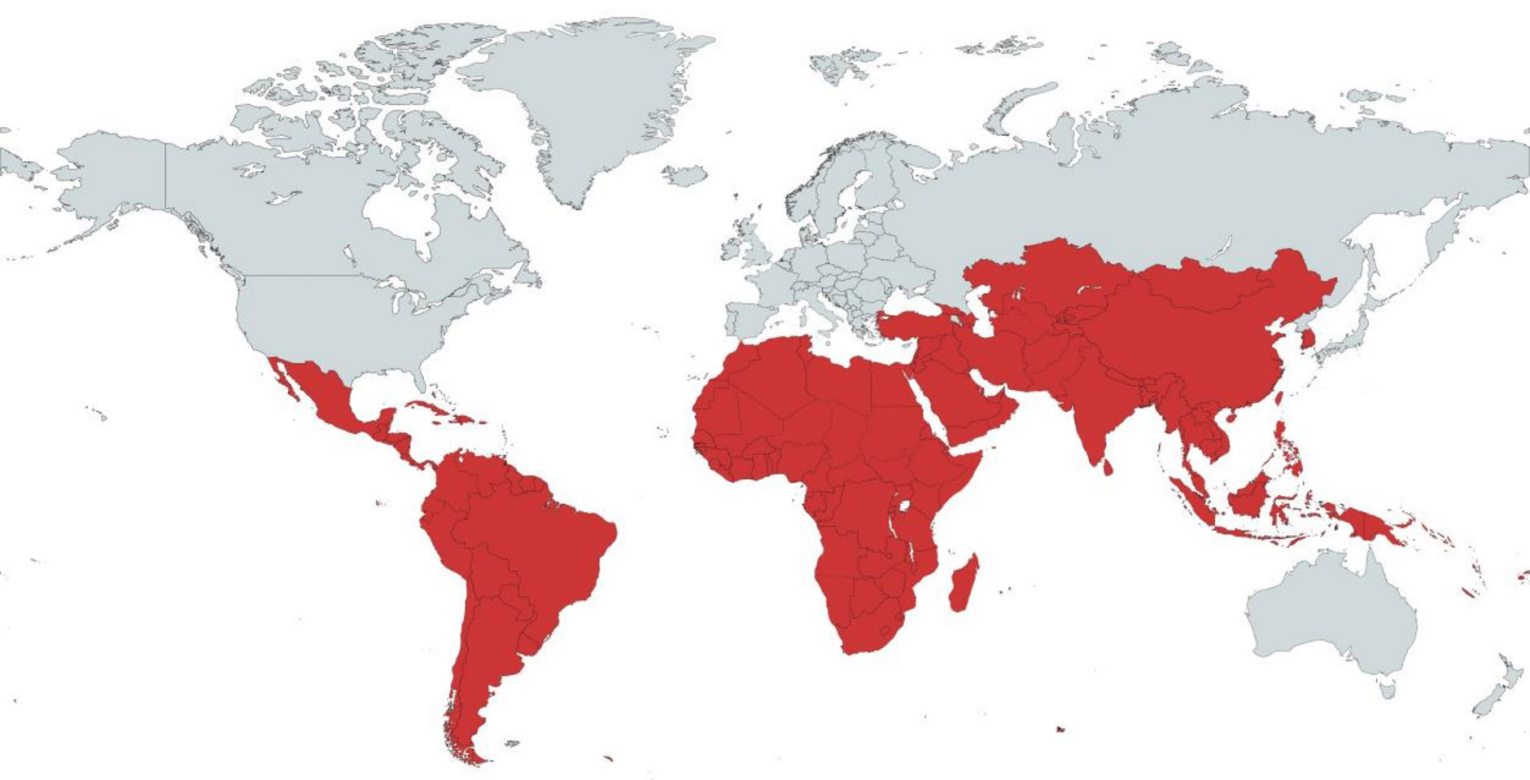
This map displays in red the LMICs that were eligible for inclusion in this study based on the United Nations classification by income and development.^15^ LMICs, low-income and middle-income countries.

Following the removal of duplicate records, the remaining titles and abstracts were screened independently by two reviewers for inclusion for full-text review. Disagreements were settled by a senior researcher. Articles were included if they reported research conducted in a LMIC between the years 2000 and 2020. LMICs were defined according to the United Nations (UN) Country Classification.^15^ The map in figure 1 illustrates the countries eligible for inclusion. Studies written in languages other than English, and studies describing aspects of HSV-2 other than interactions with HIV (priority area 1), control measures (priority area 2) or mathematical modelling (priority area 3), were excluded. Due to a relative shortage of primary research in priority areas 1 and 3, both primary and secondary researches were included, whereas there was a wealth of primary data for priority 2, and therefore, secondary data research was excluded. The systematic search was complemented by a supplementary manual scoping of reference lists of the selected studies for further articles that were not captured by the search terms. Additionally, the works of the eight key authors (Jérôme Legoff, Richard Hayes, Helen Weiss, Connie Celum, Esther Freeman, Nicolas Nagot, Katharine Looker and Jared Baeten) who had published the greatest number of individual articles from our included studies were systematically searched.

Using content analysis, information that addressed the research outcomes and the previously described quality indicators were manually extracted from the included studies and organised into a Microsoft Excel spreadsheet. The data were subsequently imported to IBM SPSS V.26 statistical software where they were cleaned and reorganised to undergo simple summary statistical analysis. Short summaries of the study outcomes were also made and assessed for similarities and differences between studies in each thematic area.

### Patient and public Involvement

Patient and public involvement was not appropriate for this study as no new patient data were collected.

## Results

**Figure 2:**
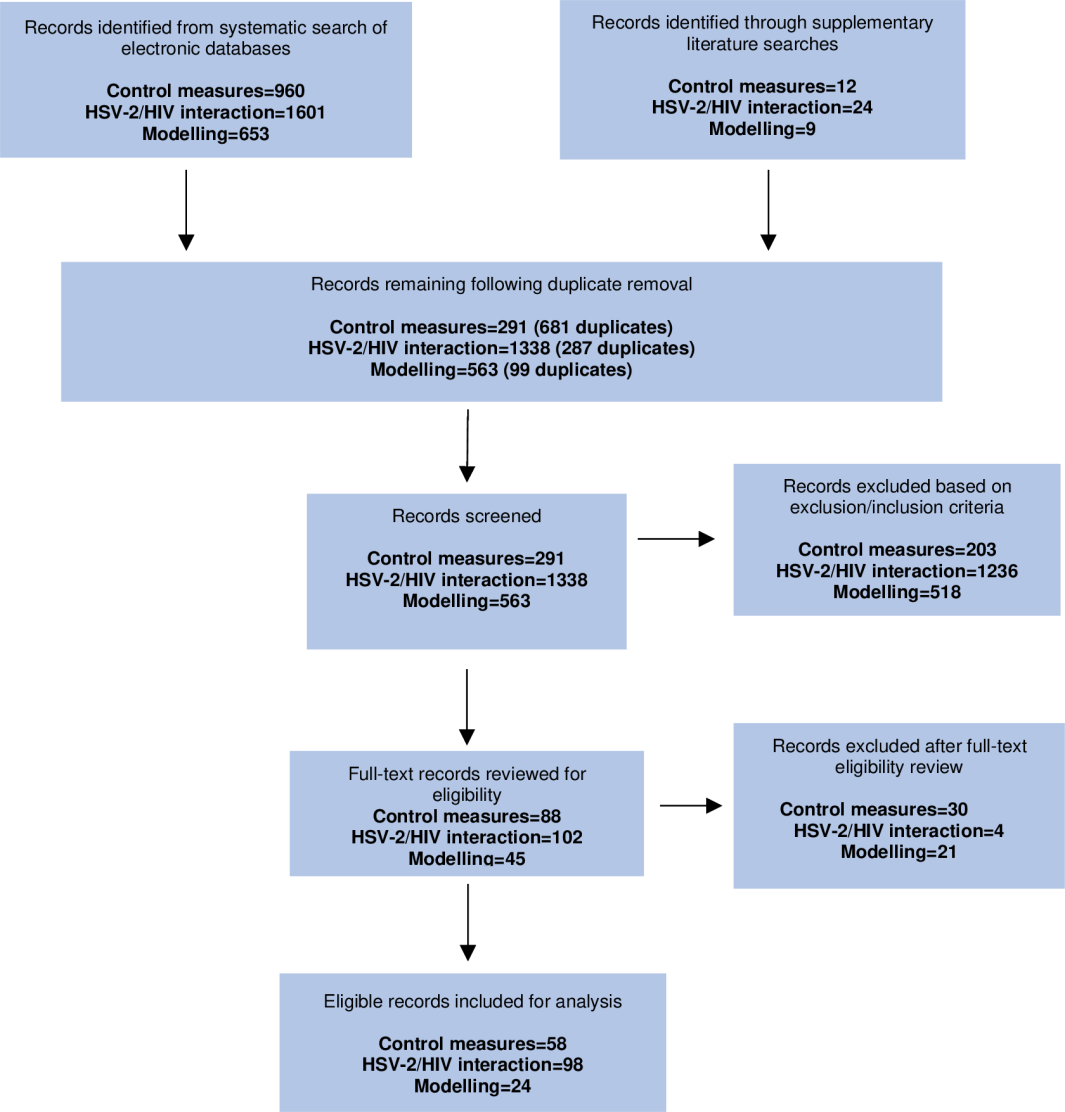
Flow chart showing study selection process for HSV-2 control measures, HSV-2/HIV interactions and HSV-2 mathematical modelling. See online supplemental appendix 3 for description of reasons for exclusion after full-text eligibility review for each research area.

A combined total of 3214 literature records were identified from the 3 electronic database searches, and 45 additional records were retrieved through supplementary search strategies. The removal of duplicates excluded 1067 of these records, which left 2192 records to manually screen. Of these records, 235 were deemed eligible for full-text review against the criteria outlined in the methods. A final total of 180 eligible online records were included for content analysis. A full list of the included studies can be viewed in online supplemental appendix 2. There was high level of screening concordance (>99%) between reviewers for the three searches. A flow diagram of the literature selection for the three research areas is presented in figure 2.

10.1136/bmjgh-2024-015167.supp2 Supplementary data

10.1136/bmjgh-2024-015167.supp3 Supplementary data

### HSV-2/HIV interactions

A total of 98 eligible studies examining the interaction between HSV-2 and HIV in LMICs between 2000 and 2020 were identified. The most commonly studied topic was the association between seroprevalences of HSV-2 and HIV (n=32, 33%), followed by the effect of HSV-2 control measures on HIV indicators (n=24, 24%), the effect of HSV-2 on HIV acquisition (n=14, 14%) and the effect of HSV-2 on HIV shedding and transmission (n=13, 13%). All other study topics (effect of HSV-2 on the clinical course of HIV, effect of HIV therapy on HSV-2 indicators, diagnostic techniques in HSV-2 and HIV-coinfected people, in vitro studies) included fewer than 10 studies. Geographically, the most common study locations were East Africa (n=44, 45%), Southern Africa (n=24, 24%) and West Africa (n=17, 17%). A total of nine studies were based in South America and South Asia, respectively (9%), six in Central Africa (6%), four in East Asia (4%) and no studies from North Africa, Central America or Western Asia. Specific study participants included people living with HIV (n=26, 27%), the general population (n=23, 23%), people living with HIV and HSV-2 (n=15, 15%), clinic attendees with presumed genital ulcer disease (GUD) (n=9, 9%), commercial sex workers (n=7, 7%), pregnant women (n=5, 5%), MSM (n=5, 5%), adolescents (n=2, 2%), HSV-2-seropositive (HIV-negative) persons (n=2, 2%) and market vendors (n=1, 1%). A total of 43% (n=42) of the studies included only female participants, 39% (n=38) included male and female participants and just 10% (n=10) included only male participants.

#### Association between HSV-2 and HIV prevalence

13 studies reported HSV-2 prevalence as being substantially higher in HIV-seropositive individuals compared with HIV seronegative.^2 16–27^ When reported as ORs, the odds of HSV-2 seropositivity in HIV-seropositive individuals was reported as varying between 4.0 and 7.9 times the odds in the HIV-seronegative individuals among different populations.^28 29^ Conversely, the odds of HIV seropositivity in HSV-2-infected people was also increased compared with the odds in HSV-2-seronegative people, although with a smaller effect size (OR=2.7).^19^ Only one study (based in Iran) reported no association between HSV-2 and HIV prevalence.^30^

#### Effect of HSV-2 on HIV indicators

Nine studies reported that HSV-2/HIV coinfection increased genital HIV shedding and/or transmission,^8 9 31–37^ while only one study found no association between coinfection and HIV shedding and/or transmission.^38^ More recently published studies recognised shedding and transmission as discrete outcomes.^9^ A 4-year prospective study of 174 HIV serodifferent couples in Uganda found that the rate of HIV transmission was greater in those with genital ulceration, compared with those without GUD (adjusted rate ratio 2.58 (95% CI, 1.03 to 5.69)). However, HIV transmission was not affected specifically by HSV-2 serological diagnosis.^39^

In terms of HIV acquisition, 10 studies agreed that HSV-2 seropositivity was associated with an increased risk of HIV acquisition.^7 40–48^ Six of these found the association to be stronger for incident HSV-2 compared with prevalent HSV-2.^7 40 42 46–48^ For example, Reynolds *et al* reported a HR of HIV acquisition of 1.67 and 3.81 for prevalent and incident HSV-2, respectively.^40^ One study in Mozambique found no association between HSV-2 and HIV acquisition.^49^ Freeman *et al* suggested that HSV-2 has a greater effect on HIV transmission than acquisition.^41^

Given the possible involvement of HSV-2 in enhancing HIV transmission/acquisition, researchers proceeded to investigate the impact of HSV-2 control measures on HIV indicators through clinical trials. Of these, eight studies found long-term HSV-2 suppressive therapy in coinfected individuals to be associated with reduced plasma and/or genital HIV levels,^50–57^ while five studies reported no significant reduction in HIV shedding with the use of suppressive therapy.^50 58–61^ Only one of these studies assessed both shedding and subsequent sexual transmission in HIV-discordant couples, and although it reported HSV-2 suppressive therapy to reduce HIV shedding, there was no evident effect on transmission.^55^ Conversely, two studies assessed the effect of HSV-2 antiviral therapy on the risk of HIV acquisition in HSV-2-seronegative individuals. Both studies found aciclovir to have no significant effect on HIV acquisition.^62 63^ The only study assessing the impact of a potential HSV-2 vaccine on HIV indicators estimated that a vaccine against HSV-2 could have a substantial effect on HIV incidence in LMIC.^64^

Population attributable fraction (PAF) of HSV-2 to HIV (the percentage of HIV cases attributable to HSV-2 infection) was reported in a total of six studies.^41 42 47 65–67^ They all estimated that a substantial proportion of HIV cases were attributable to HSV-2 with PAFs varying from 22% to 70% in different geographical populations.

#### Effect of HIV infection on HSV-2 indicators

A singe study of HIV serodiscordant couples with at least one HSV-2-seropositive partner reported HSV-2 incidence to be 2.5 times higher in partners living with HIV compared with HIV-negative people.^68^ Other studies found that tenofovir disoproxil and emtricitabine-based daily pre-exposure prophylaxis for HIV (PrEP) was associated with reduced HSV-2 incidence (as well as reduced HIV incidence),^69^ and antiretroviral therapy (ART) reduced HSV-2 shedding (as well as HIV shedding).^70^ Compared with HIV-seronegative people, people living with HIV were found to suffer more frequent and more severe HSV-2 recurrences.^60 71^

Table 1 displays the extent of the research progress according to the associated research priorities set in the 2001 WHO HSV-2 workshop.

**Table 1:** Progress towards 2001 WHO research priorities: HSV-2 and HIV interactions

2001 WHO research priority	Has the priority been addressed?			Explanation
	Yes	Partially	No	
Effect of aciclovir antiviral on HIV viral load and genital shedding	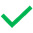			15 studies in total Viral load and genital shedding studied Multiple geographical areas studied
Differences in HIV genital shedding during primary clinical HSV-2 episodes, subclinical recurrences and in-between recurrences			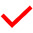	0 studies comparing shedding in the different HSV-2 disease stages
Differences in HIV viral shedding between genital lesions and from semen		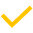		Independent studies carried out for shedding from genital lesions and semen, but no studies comparing both directly
Geographical differences in the effect of HSV-2 on HIV between Africa and other LMICs		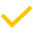		46 studies on the effect of HSV-2 on HIV; however, only 2 of these from outside of Africa No single study comparing effect of HSV-2 on HIV in Africa and another geographical region
Risk ratios/relative risk and PAF by age and sex, as well as the difference in these measures for incident and prevalent cases. More prospective studies would help achieve this		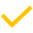		Six studies studying PAF, 2 of these divided by sex, none divided by age Risk measures reported in >15 studies, many compared risk for incident and prevalent cases 41% of the studies were prospective
Studies involving HIV-discordant couples where at least one partner is HSV-2 positive to investigate the effect of HSV-1 on transmission and acquisition		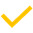		One study included HIV-discordant and HSV-seropositive couples
More information on estimated PAFs of HSV-2 for HIV		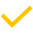		Six studies included PAF of HSV-2 for HIV as an outcome

HSV-2, herpes simplex virus type 2; LMICs, low-income and middle-income countries; PAF, population attributable fraction.

### HSV-2 control measures

A total of 58 studies were identified to address HSV-2 control measures, the most common study location being sub-Saharan Africa (n=24, 41%). The most frequently studied topic area was the use of antiviral therapy (n=27, 47%), followed by condom use (n=12, 21%), vaccine trials (n=10, 17%), microbicide use (n=6, 10%) and male circumcision (n=3, <1%). They encompassed a range of study designs including randomised control trials (n=26, 45%), literature reviews (n=13, 22%), laboratory studies (n=10, 17%), systematic reviews with meta-analyses (n=5, 9%) and cohort or cross-sectional studies (n=3, <1%).

#### Suppressive therapy

Six articles including a multiregion randomised placebo-controlled trial (RCT) among HSV-2-seropositive MSM and women in sub-Saharan Africa and South America found that suppressive therapy (400 mg twice daily aciclovir) reduced HSV-2 shedding and transmission rates.^50 57 58 72–74^ For example, Fuchs *et al* reported a 63% reduction in the frequency of genital ulcers with detectable HSV-2 and a 47% reduction in the rate of GUD recurrence overall.^72^ Analogous results were seen among male and female participants living with HIV.^58^ RCTs based in South and West Africa reported evidence to suggest that suppressive aciclovir may also be efficacious in reducing HIV shedding and disease progression.^54 58^ However, the results of further East African trials disputed this by showing no significant difference in genital and plasma HIV RNA between the control and intervention (suppressive aciclovir) arms^61^ and that suppressive aciclovir had no impact on the incidence/acquisition of HIV in HSV-2-seropositive women.^62^

#### Episodic therapy

A total of four studies investigated the effect of episodic aciclovir therapy on HSV-2 (and HIV in some instances).^60 75–77^ Episodic therapy was defined as a 5-day course of aciclovir, and the dose varied between studies from a total of 1.2 g to 1.6 g per day. Ulcer healing was the primary outcome for all four studies. Three of the four studies reported reduced ulcer duration with the use of episodic therapy, although a modest difference was reported by Baeten *et al*.^60 75 77^ On the contrary, Phiri *et al* reported no significant difference in ulcer healing with the use of aciclovir; however, this study used a relatively long duration of 14 days to assess for the presence of ulcers.^76^ HIV indicators were described as secondary outcomes in three of these studies.^60 76 77^ Reduced HIV shedding from ulcers was reported by all three studies on day 7; however, no study found a significant reduction in cervicovaginal or plasma HIV RNA.^60 76 77^

A limited number of studies (n=2) were found to evaluate the extent of HSV-2 resistance to antivirals in LMICs and the clinical benefit of early drug initiation following the onset of an ulcer lesion.^78 79^ Two studies evaluated the effect of ART in reducing HSV-2 shedding in dually infected people, with both reporting a reduction in HSV-2 shedding and acquisition, respectively.^70 79^

#### Vaccine research

Eight of the selected studies focused on the status of the HSV-2 vaccine trials and those currently under development.^80–87^ Three of these were literature reviews of the progress and challenges of developing HSV-2 vaccine,^80 81 84^ four were preclinical in vivo studies of specific candidate vaccines in mice or guinea pigs,^83 85–87^ and one was a mathematical modelling study of potential delivery methods of a theoretical vaccine.^82^ Of the vaccines tested in the preclinical studies, two were glycoprotein subunit vaccines,^83 87^ one was a live attenuated recombinant vaccine (AD472),^86^ and the other was an infected cell protein 0 (ICP0) vaccine.^85^ Both the glycoprotein subunit vaccines were found to be effective compared with the control group; however, the ICP0 vaccine showed between 10 and 100 times greater protection against HSV-2 compared with a glycoprotein subunit vaccine.^85^

Two strategies are being studied for an HSV-2 vaccine: a preventive vaccine that aims to offer protection against genital HSV-2 prior to exposure and a therapeutic vaccine that aims to reduce genital lesions and genital shedding in HSV-2-seropositive individuals. No effective vaccine is currently available for HSV-2 infection; however, six identified studies indicated that several candidate HSV-2 vaccines are in various phases of development in high-income countries (HICs). Neither of the two types of vaccines have been tested in LMICs. Despite this, there is much advocacy for the design of an HSV-2 vaccine that will be effective in LMICs.^88^ The most common products utilised for HSV-2 vaccines in clinical trials are glycoprotein subunit vaccines which work by stimulating mucosal immunity to prevent genital infection.

#### Other preventative strategies

A total of 12 articles (primary research, n=7; literature reviews, n=5) examined the impact of condom promotion or distribution in reducing HSV-2 acquisition.^73 89–99^ Of the interventional research studies, four studies (n=4, 44%) reported condom use as having no significant effect on HSV-2 acquisition,^91 93 96 97^ whereas three studies (n=3, 33%) reported a statistically significant reduction in HSV-2 acquisition.^73 92 98^

The potential effect of topical microbicides in controlling HSV-2 spread was the focus of five studies.^100–104^ Although no safe and effective microbicide has yet been approved for the prevention of HSV-2 transmission, numerous candidates showed encouraging results. All the microbicides investigated targeted broad categories of STIs, including HSV-2. The 2001 WHO workshop listed several potential topical microbicides for combatting HSV-2 infection, including Nonoxynol-9 and pH-modifying products (Buffergel).^10^ Following the result of the phase 2 clinical trials in Africa, Nonoxynol-9 was found to offer no protection against HIV and actually increased the risk of genital disease.^102^

Only three of the identified articles analysed the effect of male circumcision on HSV-2.^42 105 106^ For two of these studies, the primary outcome was the impact of male circumcision on HIV indicators, and HSV-2 was a secondary research outcome.^42 106^ Both Yuan *et al* and Tobian *et al* reported that male circumcision reduced the risk of HSV-2 seropositivity,^105 106^ while Sobngwi-Tambekou *et al* concluded that HSV-2 seropositivity did not have an impact on the protective effects of male circumcision on HIV acquisition.^42^

Table 2 displays the extent of the research progress according to the associated research priorities set in the 2001 WHO HSV-2 workshop.

**Table 2:** Progress towards 2001 WHO research priorities: HSV-2 control measures

2001 WHO research priority: control measures	Has the priority been addressed?			Explanation
	Yes	Partially	No	
Development of African reference laboratories to monitor aciclovir resistance		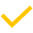		Only one study (literature review) focusing on aciclovir resistance^78^
Evaluation of the current syndromic management of GUD in areas of high HSV-2 prevalence (whether to add aciclovir)		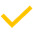		Two studies evaluating the syndromic management of HSV-2 episodes^76 77^
Monitoring of the clinical/cost-effectiveness of aciclovir use in the syndromic management of GUD			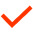	No studies evaluating the cost effectiveness of episodic aciclovir as part of the syndromic approach
Trials of episodic therapy, measuring the effect on HIV shedding and HSV-2 shedding	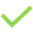			Three clinical trials measured both HSV-2 and HIV shedding as an outcome^60 76 77^
Trials of episodic therapy, measuring the effect on HIV acquisition or transmission			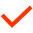	No studies evaluating the effect of episodic HSV-2 therapy on HIV acquisition
Trials of suppressive therapy in high-risk groups, measuring the effect on HIV acquisition among individuals at high risk of infection		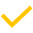		Only one of the HSV-2 suppressive therapy studies included HIV acquisition as a research outcome^62^
Lobby for investment in prophylactic vaccine development		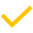		Modest amount of research development in relation to prophylactic vaccine development
Develop locally administered vaccines			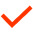	No studies published on locally administered vaccines for HSV-2
Evaluate counselling strategies for HSV-2-seropositive individuals			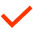	No identified studies on counselling for HSV-2-seropositive individuals
Include HSV-2 as an outcome in microbicide trials	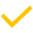			Five studies evaluated the effect of topical microbicides on HSV-2, as well as other STIs^100–104^

GUD, genital ulcer disease; HSV-2, herpes simplex virus type 2; STIs, sexually transmitted infections.

### Mathematical modelling

A total of 24 eligible studies examining HSV-2 mathematical were identified. The most common study topics included the modelling of HSV-2 and HIV interactions (n=10, 42%) and the modelling of HSV-2 control measures (n=8, 33%). Other topic areas included modelling of HSV-2 transmission (n=5, 21%), modelling the burden of HSV-2 (n=2, 8%) and improving HSV-2 modelling techniques (n=2, 10%). The majority of the studies were based on data from East Africa (n=11, 45.8%); nine were from Southern Africa (42.9%) and four from both West Africa and South Asia (16.7%). The two most commonly used models in the studies were the STDSIM and Markov models (dynamic stochastic models).^107 108^ Both the studies that were aimed at further developing HSV-2 mathematical modelling capabilities concluded that existing models could be simplified without a significant loss of accuracy in order to aid their interpretation.^108 109^

Incorporating aciclovir into the syndromic management of HSV-2 was predicted to be a cost-effective strategy in reducing HSV-2 burden in LMIC areas with high prevalence.^110^ A South African study estimated that female-to-male sexual transmission of HSV-2 could be reduced by male circumcision,^31^ and schooling interventions were also predicted to reduce HSV-2 risk through its impact on future aspirations and likelihood of having sex.^111^ Tenofovir disoproxil vaginal microbicide was predicted to cause only a 4.9% reduction in HSV-2 incidence 15 years after its introduction,^112^ and the protective effects of tenofovir DF/emtricitabine HIV PrEP against HSV-2 were not sufficient as to enhance its overall cost-effectiveness.^113^ Studies modelling the impact of HSV-2 therapy (non-vaccine) on HIV indicators predicted modest results, with limitations including cost-effectiveness, and specific unrealistic assumptions underlying the conclusions.^109 114 115^ Studies modelling vaccine impact were few. It was predicted in two separate studies that an HSV-2 vaccine could positively impact both HSV-2 and HIV incidence in LMICs.^64^

Variations in HSV-2 sexual transmission rates were largely explained by sexual network characteristics such as marital status and number of partners.^116 117^ Among heterosexual married couples with HSV-2 infection, it was estimated that the virus was introduced by the male partner via sexual activity outside of marriage in 64.1% of cases.^118^ These studies were conducted to aid the targeting of interventions.

Table 3 displays the extent of the research progress according to the associated research priorities set in the 2001 WHO HSV-2 workshop.

**Table 3:** Progress towards 2001 WHO research priorities: HSV-2 mathematical modelling

2001 WHO research priority: mathematical modelling	Has the priority been addressed?			Explanation
	Yes	Partially	No	
Further models of HSV-2 transmission, control measures and interaction with HIV	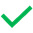			Multiple studies carried out for HSV-2 transmission (3), control measures (5) and interaction with HIV (9)
Modelling risk of antiviral resistance			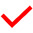	0 studies on modelling the risk of antiviral resistance; however, there were non-modelling studies on aciclovir resistance
Modelling the cost-effectiveness of specific control measures		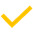		Two studies carried out on cost-effectiveness of control measures, further analysis recommended
Modelling the potential effect of a HSV-2 vaccine		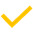		Two studies carried out to model the effect of a vaccine, many more studies emphasised its importance

HSV-2, herpes simplex virus type 2.

## Discussion

This review employed a content analysis methodology to describe the progress made in meeting the 2001 WHO research priorities for HSV-2/HIV interactions, HSV-2 control and mathematical modelling.^12^

### Successfully addressed WHO HSV-2 priorities

The published research over the past 20 years in the areas of HSV-2/HIV interactions, HSV-2 control measures and mathematical modelling has certainly reflected the WHO research priorities set in 2001,^12^ with most of the identified studies addressing at least one of the documented priorities. The greatest number of the included studies were related to the interaction between HSV-2 and HIV; this was expected since the prospect of harnessing this relationship to reduce the burden of HIV has been a key driver of HSV-2 research over this period.^119^

All but one of the seven priorities for HSV-2 and HIV interactions were addressed in the literature. A considerable amount of research attention was given to the effect of HSV-2 therapeutics on HIV shedding and viral load. However, many of these studies extrapolated a positive link between HSV-2 therapeutics and reduced HIV shedding and/or viral load to inferring a link between HSV-2 therapeutics and reduced HIV transmission. Only two studies explored the effect of HSV-2 therapeutics on both HIV shedding and sexual transmission. The studies found that although aciclovir reduced HIV genital shedding in coinfected people, it did not result in a corresponding reduction in the rate of forward transmission.^55^ This highlights both the importance of avoiding this overinterpretation and the need for more studies to extend their outcome measures to include HIV sexual transmission.

A total of 52 studies on HSV-2 control measures were published within the selected time frame, and they addressed 6 of the 10 research priorities that were outlined at the WHO priority meeting. Most of the identified studies revealed that, currently, the two best-established and recommended prevention methods for HSV-2 infection are behavioural intervention and HSV-2 antiviral treatment.

A relatively small number (n=20) of mathematical modelling studies were published in the studied time frame; however, they did address three out of four of the 2001 research priorities. Although HSV-2 was found to have had a substantial bearing on the HIV epidemic and multiple authors emphasising the need for a vaccine, very few studies were based on a potential HSV-2 vaccine.

### Unaddressed priorities and research gaps

The majority of unaddressed WHO priorities involved HSV-2 control measures (n=4). Unaddressed priorities from 2001 were identified across the three priority areas. These included the monitoring of the clinical/cost-effectiveness of aciclovir in GUD syndromic management, trials of episodic therapy measuring the effect on HIV acquisition or transmission, evaluation of counselling strategies and the development of locally administered vaccines. Although there were studies assessing the clinical effectiveness of aciclovir in the syndromic approach to GUD, none of these included cost-effectiveness as an outcome. This outcome measure would have been useful supporting evidence for developing GUD syndromic management in LMICs, where the use of aciclovir is much less frequent than in HICs. The impact of HSV-2 control measures on HIV indicators was frequently cited; however, this was always in the context of suppressive therapy, rather than episodic therapy. This may have been due to the more complex study design that would be required to investigate the effect of episodic therapy on HSV-2 shedding over multiple HSV-2 episodes.

The last two unaddressed priorities were the exploration of the differences in HIV genital shedding during primary clinical HSV-2 episodes, subclinical recurrences and in-between recurrences and the modelling of the risk of antiviral resistance. Antiviral resistance may not have been deemed a priority in LMIC during the studied time period because even by 2010 numerous African countries were yet to incorporate aciclovir into their GUD syndromic management algorithm, despite its patent expiring in the late 1990s.^120^ It is therefore unlikely that researchers could justify the study of aciclovir resistance when the drug was rarely used due to relative unavailability. Indeed, aciclovir resistance testing is not even available in many HICs.^121^ No studies were identified to address counselling strategies which may be owing to the high number of hours and skilled personnel required for the intervention.

### HSV-2 vaccine

Despite consistent emphasis on the urgent need to focus on vaccine research and development, the progress has been poor in this respect. A possible reason for modest progress may be the relatively small size of the vaccine industry compared with the market for curative therapy which patients consume daily.^122^ There have been concerns for some time about disinvestment in research of vaccines because of their relative lack of profitability for pharmaceutical companies.^123^ Alternatively, this may be attributable to publication bias whereby studies obtaining negative results were not published. Nevertheless, the identified studies obtained encouraging results highlighting satisfactory cost-effectiveness and improvements in both HSV-2 and HIV indicators.^64 117^

In vitro vaccine studies conducted in HIC to develop vaccines for use in LMIC would not have been included, for example, Morello *et al*.^124^

### Impact of the 2001 WHO workshop

Only one of the 180 included studies in this review cited the 2001 WHO HSV-2 workshop report in their work.^125^ However, many of the workshop attendees proceeded to publish research that addressed the priorities in the 2001 workshop report. The eight key authors listed in the methodology were responsible for 61% (n=111) of the included articles in this study, and many had collaborated in their research. Of these, 38% attended the workshop. This may therefore have influenced a notable proportion of the subsequent research.

### Limitations

There are limitations to the analytical approach of this study that increase the risk of bias. First, although conducting the content analysis manually allowed researcher immersion in the data, this increased the risk of unconscious cognitive biases such as confirmation and attribution bias. This may have affected the interpretation of the included papers and hence how they were categorised and reported. Additionally, this study did not include an in-depth appraisal of the strengths and limitations of all the identified studies; therefore, the description of the studies’ main findings should be interpreted with caution. Further, although the research was conducted by a team of experienced clinical and research experts, a more holistic approach would have included a wider range of stakeholders, particularly from LMICs.

The search strategy also had its limitations. Due to the time and resource constraints, separate search terms for each developing country could not be used, although the search was conducted with the support of an experienced librarian. Also, EMBASE, a commonly used database, was not included in the search strategy because the host institutions did not have access to it. Finally, only English language studies were included. These limitations meant that relevant studies written in languages other than English will have been excluded. Additionally, some relevant literature within the EMBASE database and additional studies that could have been identified through including individual LMIC names in the search strategies will likely have been missed. This may particularly affect research published in French since it is the national language of many West and Central African countries. Unpublished research could also not be included which therefore risks publication bias. Finally, because a number of the vaccine studies were laboratory based and did not include any participants, studies that were conducted in HIC (exclusion criteria) with the ultimate aim of use in LMIC would not have been included, for example, Morello *et al*.^124^ Nevertheless, our systematic content analysis of so broad a range of HSV-2 research, over a 20-year period, provides information that may help inform future priority setting.

Future HSV-2 priority setting: issues for consideration in future HSV-2-related priority setting for each respective research area include the following.

#### HSV-2/HIV interactions

There was a relative lack of studies assessing the effect of HSV-2 on HIV shedding that included HIV transmission as an outcome measure. While in the past years, this paucity would have shaped future priority setting, addressing this research gap with the current knowledge of U=U (HIV undetectable = untransmittable) would be unethical.^126^ This exemplifies the importance of reviewing research priorities in dynamic research fields where new knowledge can significantly alter the landscape.

#### HSV-2 control measures

- Further research on HSV-2 control measures involving young adults, particularly women, as an increased HSV-2 prevalence rate was noted in this age stratum.
- Further assessment of microbicide candidates as many of them showed promising initial results. Further focus to be placed on evaluating the microbicide effect in terms of preventing HSV-2 infection in HSV-2 discordant couples.
- Although few studies reported encouraging results for either HSV-2 vaccination type in laboratory-based studies in developed countries, no research of this nature was identified based in developing countries. Therefore, further HSV-2 vaccine research is needed in LMICs. The public health gains of a successful vaccine, given the high rates of acquisition in adolescence (e.g in Africa), could be substantial.

#### HSV-2 mathematical modelling

- Further research modelling the impact of HSV-2 vaccination on HSV-2 and HIV indicators which better reflects disease burden
- Further studies to model the cost-effectiveness of proposed interventions to better inform future programmes
- Further research modelling the risk of antiviral resistance, since an increasing number of LMICs are incorporating aciclovir into the syndromic management algorithm for GUD

## Conclusion

In summary, the research carried out between 2000 and 2020 in the areas of HSV-2 and HIV interactions, HSV-2 control measures and HSV-2 mathematical modelling has largely reflected the priorities set in the 2001 WHO HSV-2 workshop. Being the principal driver of HSV-2 research during this time, the most studied topic was the interaction between HSV-2 and HIV, and the most frequently studied geographical area was East Africa.

However, notable research gaps remain, in particular, in HSV-2 vaccine research, antiviral resistance and monitoring of the clinical/cost-effectiveness of aciclovir use in the syndromic management of GUD. Updated research priorities for HSV-2 may focus on increasing the number of vaccine and microbicide studies conducted in LMICs and prospective studies of HIV-discordant couples with HSV-2 and exploring neglected geographical areas such as North Africa and the Middle East.

## Data Availability

All data relevant to the study are included in the article or uploaded as supplementary information.
