# Supplementary material for: 20 years of herpes simplex virus type 2 (HSV-2) research in low-income and middle-income countries: systematic evaluation of progress made in addressing WHO priorities for research in HSV-2/HIV interactions, HSV-2 control and mathematical modelling
Source: BMJ Glob Health. 2024 Jul 4;9(7):e015167. doi: 10.1136/bmjgh-2024-015167 (PMC11227757; doi:10.1136/bmjgh-2024-015167)
Supplement: Supplementary data [file bmjgh-2024-015167supp001.pdf]

Appendix 1 – Search strategy **(MEDLINE, CINAHL, Global Health, Cochrane)**

Objective 1- HSV-2 control measures

**Database: MEDLINE**		
**Date searched: 01/08/2020**		
**Search number**	**Search terms**	**Number of results**
S10	S3 AND S6 AND S9	211
S9	S7 OR S8	289,280
S8	(MH "Developing Countries")	74,612
S7	“developing countr*” OR “low income countr*” OR LIC OR LMIC OR “low to middle income countr*” OR “low-to-middle income countr*” OR “low-tomiddle income” OR “low to middle income” OR “low income” OR “middle income” OR “least developed countr*” OR “less developed countr*” OR “under developed countr*” OR “under developed nation*” OR “poor countr*” OR “third world countr*” OR “third world nation*” OR “least developed nation*” OR “less developed nation*” OR “global south” OR “sub-Sah	289,280
S6	S4 OR S5	2,412,767
S5	(MH "Primary Prevention+") OR (MH "Secondary Prevention") OR (MH "Tertiary Prevention") OR (MH "Vaccines+") OR (MH "Case Management")OR (MH "Antiviral Agents+") OR (MH "Acyclovir+") OR (MH "Condoms")	490,269
S4	“control measure” OR “preventive measure” OR “intervention” OR “benefit” OR “protection genital herpes” OR “condom” OR “vaccine” OR “case management” OR “Episodic therapy” OR “suppressive therapy” OR “Antiviral drugs” OR “Aciclovir* OR “acyclovir resistance” OR “resistance” OR “behavioural interventions” OR “suppressive”	2,203,422
S3	S1 OR S2	81,850
S2	(MH "Herpes Genitalis") OR (MH "Herpesvirus 2, Human") OR (MH "Herpes Simplex+") OR (MH "Herpes Labialis")	25,607

S1	“HSV-2” OR “genital herpes” OR “herpes” OR “herpes genitalis” OR “herpes simplex 2” OR “human simplex virus 2” OR “herpes virus 2”	79,564

Database: CINAHL		
**Date searched: 01/08/2020**		
**Search number**	**Search terms**	**Number of results**
S10	S3 AND S6 AND S9	82
S9	S7 OR S8	82,345
S8	(MH "Developing Countries")	19,539
S7	“developing countr*” OR “low income countr*” OR LIC OR LMIC OR “low to middle income countr*” OR “low-to-middle income countr*” OR “low-tomiddle income” OR “low to middle income” OR “low income” OR “middle income” OR “least developed countr*” OR “less developed countr*” OR “under developed countr*” OR “under developed nation*” OR “poor countr*” OR “third world countr*” OR “third world nation*” OR “least developed nation*” OR “less developed nation*” OR “global south” OR “sub-Sahharan Africa” OR “Asia” OR “South America”	82,345
S6	S4 OR S5	676,146
S5	(MH "Preventive") OR (MH "Vaccines+") OR (MH "Case Management") OR (MH "Antiviral Agents+") OR (MH "Acyclovir+") OR (MH "Condoms+")	122,709
S4	“control measure” OR “preventive measure” OR “intervention” OR “benefit” OR “protection genital herpes” OR “condom” OR “vaccine” OR “case management” OR “Episodic therapy” OR “suppressive therapy” OR “Antiviral drugs” OR “Aciclovir* OR “acyclovir resistance” OR “resistance” OR “behavioural interventions” OR “suppressive”	636,087
S3	S1 OR S2	15,035
S2	(MH "Herpesviruses+") OR (MH "Herpes Genitalis")	7,177

S1	"HSV-2" OR "genital herpes" OR "herpes" OR "herpes genitalis" OR "herpes simplex 2" OR "human simplex virus 2" OR "herpes virus 2"	11,345

**Database: Global Health**		
**Date searched: 01/08/2020**		
**Search number**	**Search terms**	**Number of results**
S10	S3 AND S6 AND S9	623
S9	S7 OR S8	855,928
S8	DE "Developing Countries" OR DE "Least Developed Countries"	843,726
S7	"developing countr*" OR "low income countr*" OR LIC OR LMIC OR "low to middle income countr*" OR "low-to-middle income countr*" OR "low-tomiddle income" OR "low to middle income" OR "low income" OR "middle income" OR "least developed countr*" OR "less developed countr*" OR "under developed countr*" OR "under developed nation*" OR "poor countr*" OR "third world countr*" OR "third world nation*" OR "least developed nation*" OR "less developed nation*" OR "global south" OR "sub-Sahharan Africa" OR "Asia" OR "South America"	855,682
S6	S4 OR S5	582,283
S5	DE "prevention" OR "vaccines" OR DE "antiviral agents" OR DE "condoms"	157,308
S4	"control measure" OR "preventive measure" OR "intervention" OR "benefit" OR "protection genital herpes" OR "condom" OR "vaccine" OR "case management" OR "Episodic therapy" OR "suppressive therapy" OR "Antiviral drugs" OR "Aciclovir* OR "acyclovir resistance" OR "resistance" OR "behavioural interventions" OR "suppressive"	513,792
S3	S1 OR S2	14,030
S2	DE "Human herpesvirus 2" OR DE "herpes simplex viruses"	7,710
S1	"HSV-2" OR "genital herpes" OR "herpes" OR "herpes genitalis" OR "herpes simplex 2" OR "human simplex virus 2" OR "herpes virus 2"	13,869

**Database: Cochrane**		
**Date searched: 01/08/2020**		
**Search number**	**Search terms**	**Number of results**
S10	S3 AND S6 AND S9	44
S9	S7 OR S8	20,419
S8	[Developing Countries]	835
S7	"developing countr*" OR "low income countr*" OR LIC OR LMIC OR "low to middle income countr*" OR "low-to-middle income countr*" OR "low-tomiddle income" OR "low to middle income" OR "low income" OR "middle income" OR "least developed countr*" OR "less developed countr*" OR "under developed countr*" OR "under developed nation*" OR "poor countr*" OR "third world countr*" OR "third world nation*" OR "least developed nation*" OR "less developed nation*" OR "global south" OR "sub-Saharan Africa" OR "Asia" OR "South America"	20,419
S6	S4 OR S5	385,322
S5	[Primary Prevention] OR [Vaccines] OR [Case Management] OR [Antiviral Agents] OR [Acyclovir] OR [Condoms]	23,991
S4	"control measure" OR "preventive measure" OR "intervention" OR "benefit" OR "protection genital herpes" OR "condom" OR "vaccine" OR "case management" OR "Episodic therapy" OR "suppressive therapy" OR "Antiviral drugs" OR "Aciclovir" OR "acyclovir resistance" OR "resistance" OR "behavioural interventions" OR "suppressive"	375,679
S3	S1 OR S2	1705
S2	[Herpes Simplex] OR [Herpesvirus 2, Human] OR [Herpes Genitalis]	858
S1	"HSV-2" OR "genital herpes" OR "herpes" OR "herpes genitalis" OR "herpes simplex 2" OR "human simplex virus 2" OR "herpes virus 2"	1457

Objective 2- HSV-2 and HIV interactions

**Database: MEDLINE**		
**Date searched: 01/08/2020**		
**Search number**	**Search terms**	**Number of results**
S6	Limiters- full text only	318
S5	Limiters- English only, post year 2000	402
S4	S1 AND S2 AND S3	639
S3	(MH "diagnosis") OR (MH "polymerase chain reaction") OR (MH "cell culture techniques") OR (MH "serologic tests*") OR (MH "serology") OR (MH "immunoassay") OR diagnos* OR "diagnostic test*" OR test* OR "rapid diagnostic test*" OR "rapid test*" OR "point of care test*" OR NAAT OR "Nucleic Acid Amplification Test" OR PCR OR "Polymerase chain reaction" OR immunoassay OR culture OR serolog* OR antibod*	10,037,490
S2	(MH "developing countries") OR "developing countr*" OR LMIC OR "low to middle income" OR "low to middle income" OR "low income" OR "middle income" OR "lEast developed countr*" OR "less developed countr*" OR "under developed countr*" OR "under developed nation*" OR "poor countr*" OR "third world countr*" OR "third world nation*" OR "lEast developed nation*" OR "less developed nation*" OR "global South" OR "sub-Saharan Africa" OR Asia OR "South America"	288,482
S1	(MH "Herpes Simplex+") OR (MH "Herpes Labialis") OR (MH "Herpes Genitalis") OR (MH "Herpesvirus 2, Human") OR "herpes virus 2" OR "human simplex virus 2" OR "herpes simplex 2" OR "herpes genitalis" OR herpes OR "genital herpes" OR HSV-2	81,907

**Database: CINAHL**		
**Date searched: 01/08/2020**		
**Search number**	**Search terms**	**Number of results**
S6	Limiters- full text only	109
S5	Limiters- English only, post year 2000	116
S4	S1 AND S2 AND S3	125
S3	(MH “diagnosis”) OR (MH “polymerase chain reaction”) OR (MH “laboratory diagnosis”) OR diagnos* OR “diagnostic test*” OR test* OR “rapid diagnostic test*” OR “rapid test*” OR “point of care test*” OR NAAT OR “Nucleic Acid Amplification Test” OR PCR OR “Polymerase chain reaction” OR immunoassay OR culture OR serolog* OR antibod*	2,179,651
S2	(MH “developing countries”) OR (MH “low and middle income countries”) OR “developing countr*” OR LMIC OR “low-to-middle income” OR “low to middle income” OR “low income” OR “middle income”OR “lEast developed countr*” OR “less developed countr*” OR “under developed countr*” OR “under developed nation*” OR “poor countr*” OR “third world countr*” OR “third world nation*” OR “lEast developed nation*” OR “less developed nation*” OR “global South” OR “sub-Saharan Africa” OR Asia OR “South America”	82,099
S1	(MH "herpesviruses") OR (MH "Herpes Genitalis") OR (MH “herpes simplex”) OR “herpes virus 2” OR “human simplex virus 2” OR “herpes simplex 2” OR “herpes genitalis” OR herpes OR “genital herpes” OR HSV-2	12,567

**Database: Global Health**		
**Date searched: 01/08/2020**		
**Search number**	**Search terms**	**Number of results**
S6	Limiters- full text only	1,167
S5	Limiters- English only, post year 2000	2,047
S4	S1 AND S2 AND S3	2,350
S3	(DE “diagnosis”) OR (DE “laboratory diagnosis”) OR (DE “immunodiagnosis”) OR diagnos* OR “diagnostic test*” OR test* OR “rapid diagnostic test*” OR “rapid test*” OR “point of care test*” OR NAAT OR “Nucleic Acid Amplification Test” OR PCR OR “Polymerase chain reaction” OR immunoassay OR culture OR serolog* OR antibod*	1,100,893
S2	(DE “developing countries”) OR (DE “lEast developed countries”) OR (DE “SADC Countries”) OR “developing countr*” OR LMIC OR “low-to-middle income” OR “low to middle income” OR “low income” OR “middle income”OR “lEast developed countr*” OR “less developed countr*” OR “under developed countr*” OR “under developed nation*” OR “poor countr*” OR “third world countr*” OR “third world nation*” OR “lEast developed nation*” OR “less developed nation*” OR “global South” OR “sub-Saharan Africa” OR Asia OR “South America”	969,847
S1	(DE "human herpesvirus 2") OR (DE “Herpes Simplex Viruses”) OR “herpes virus 2” OR “human simplex virus 2” OR “herpes simplex 2” OR “herpes genitalis” OR herpes OR “genital herpes” OR HSV-2	13,918

Database: Cochrane Library		
**Date searched: 01/08/2020**		
**Search number**	**Search terms**	**Number of results**
S6	Limiters- full text only	7
S5	Limiters- English only, post year 2000	7
S4	S1 AND S2 AND S3	7
S3	(MH “diagnosis”) OR diagnos* OR “diagnostic test*” OR test* OR “rapid diagnostic test*” OR “rapid test*” OR “point of care test*” OR NAAT OR “Nucleic Acid Amplification Test” OR PCR OR “Polymerase chain reaction” OR immunoassay OR culture OR serolog* OR antibod*	437,893
S2	(MH “developing countries”) OR “developing countr*” OR LMIC OR “low-to-middle income” OR “low to middle income” OR “low income” OR “middle income”OR “lEast developed countr*” OR “less developed countr*” OR “under developed countr*” OR “under developed nation*” OR “poor countr*” OR “third world countr*” OR “third world nation*” OR “lEast developed nation*” OR “less developed nation*” OR “global South” OR “sub-Saharan Africa” OR Asia OR “South-America”	6,192
S1	(MH "human herpes virus 2") OR “herpes virus 2” OR “human simplex virus 2” OR “herpes simplex 2” OR “herpes genitalis” OR herpes OR “genital herpes” OR HSV-2	4,288

Objective 3 – HSV-2 mathematical modelling

Database: MEDLINE		
**Date searched: 02/08/2020**		
**Search number**	**Search terms**	**Number of results**
S6	Limiters- full text only	129
S5	Limiters- English only, post year 2000	130
S4	S1 AND S2 AND S3	172
S3	(MH “biological models”) OR (MH “statistical models”) OR (MH “theoretical models”) OR “mathematical model*” OR “model* OR “disease model*” OR ““statistical model*” OR “vaccine* model*” OR predict*	4,951,333
S2	(MH “developing countries”) OR “developing countr*” OR LMIC OR “low-to-middle income” OR “low to middle income” OR “low income” OR “middle income”OR “lEast developed countr*” OR “less developed countr*” OR “under developed countr*” OR “under developed nation*” OR “poor countr*” OR “third world countr*” OR “third world nation*” OR “lEast developed nation*” OR “less developed nation*” OR “global South” OR “sub-Saharan Africa” OR Asia OR “South America”	288,482

S1	(MH "Herpes Simplex+") OR (MH "Herpes Labialis") OR (MH "Herpes Genitalis") OR (MH "Herpesvirus 2, Human") OR “herpes virus 2” OR “human simplex virus 2” OR “herpes simplex 2” OR “herpes genitalis” OR herpes OR “genital herpes” OR HSV-2	81,907

**Database: CINAHL**		
**Date searched: 02/08/2020**		
**Search number**	**Search terms**	**Number of results**
S6	Limiters- full text only	27
S5	Limiters- English only, post year 2000	29
S4	S1 AND S2 AND S3	30
S3	(MH “biological models”) OR (MG “statistical models”) OR “mathematical model*” OR model* OR “disease model*” OR “statistical model*” OR “vaccin* model*” OR predict*	946,209
S2	(MH “developing countries”) OR (MH “low and middle income countries”) OR “developing countr*” OR LMIC OR “low-to-middle income” OR “low to middle income” OR “low income” OR “middle income”OR “lEast developed countr*” OR “less developed countr*” OR “under developed countr*” OR “under developed nation*” OR “poor countr*” OR “third world countr*” OR “third world nation*” OR “lEast developed nation*” OR “less developed nation*” OR “global South” OR “sub-Saharan Africa” OR Asia OR “South America”	82,099
S1	(MH "herpesviruses") OR (MH "Herpes Genitalis") OR (MH “herpes simplex”) OR “herpes virus 2” OR “human simplex virus 2” OR “herpes simplex 2” OR “herpes genitalis” OR herpes OR “genital herpes” OR HSV-2	12,567

Database: Global Health		
**Date searched: 02/08/2020**		
**Search number**	**Search terms**	**Number of results**
S6	Limiters- full text only	495
S5	Limiters- English only, post year 2000	495
S4	S1 AND S2 AND S3	523
S3	(DE “mathematical models”) OR d“mathematical model*” OR model* OR “disease model*” OR “statistical model*” OR “vaccin* model*” OR predict*	814,970
S2	(DE “developing countries”) OR (DE “lEast developed countries”) OR (DE “SADC Countries”) OR “developing countr*” OR LMIC OR “low-to-middle income” OR “low to middle income” OR “low income” OR “middle income”OR “lEast developed countr*” OR “less developed countr*” OR “under developed countr*” OR “under developed nation*” OR “poor countr*” OR “third world countr*” OR “third world nation*” OR “lEast developed nation*” OR “less developed nation*” OR “global South” OR “sub-Saharan Africa” OR Asia OR “South America”	969,847
S1	(DE "human herpesvirus 2") OR (DE “Herpes Simplex Viruses”) OR “herpes virus 2” OR “human simplex virus 2” OR “herpes simplex 2” OR “herpes genitalis” OR herpes OR “genital herpes” OR HSV-2	13,918

**Database: Cochrane Library**		
**Date searched: 02/08/2020**		
**Search number**	**Search terms**	**Number of results**
S6	Limiters- full text only	2
S5	Limiters- English only, post year 2000	2
S4	S1 AND S2 AND S3	2
S3	(MH “biological models”) OR (MG “statistical models”) OR “mathematical model*” OR model* OR “disease model*” OR “statistical model*” OR “vaccin* model*” OR predict*	112,680
S2	(MH “developing countries”) OR “developing countr*” OR LMIC OR “low-to-middle income” OR “low to middle income” OR “low income” OR “middle income”OR “lEast developed countr*” OR “less developed countr*” OR “under developed countr*” OR “under developed nation*” OR “poor countr*” OR “third world countr*” OR “third world nation*” OR “lEast developed nation*” OR “less developed nation*” OR “global South” OR “sub-Saharan Africa” OR Asia OR “South America”	6,192
S1	(MH "human herpes virus 2”) OR “herpes virus 2” OR “human simplex virus 2” OR “herpes simplex 2” OR “herpes genitalis” OR herpes OR “genital herpes” OR HSV-2	4,286
