# Supplementary material for: 20 years of herpes simplex virus type 2 (HSV-2) research in low-income and middle-income countries: systematic evaluation of progress made in addressing WHO priorities for research in HSV-2/HIV interactions, HSV-2 control and mathematical modelling
Source: BMJ Glob Health. 2024 Jul 4;9(7):e015167. doi: 10.1136/bmjgh-2024-015167 (PMC11227757; doi:10.1136/bmjgh-2024-015167)
Supplement: Supplementary data [file bmjgh-2024-015167supp002.pdf]

Appendix 2- articles included and characteristics

Control measures

| Authors | Year | Title | Geo_Area of study population | Main findings/Prevalence | Journal |
|---|---|---|---|---|---|
| Kamali, A f Quigley, M ; Nakiyingi, J ; Kinsman, J ; Kengeya-Kayondo, J ; Gopal, R ; Ojwiya, A ; Hughes, P ; Carpenter, L ; Whitworth, J. | 2003 | Syndromic management of sexually-transmitted infections and behaviour change interventions on transmission of HIV-1 in rural Uganda: a community randomised trial | East Africa | Incidence of HSV2 was lower in group A than in group C (incidence rate ratio 0·65, 0·53–0·80) however, more effective STI and behavioural interventions need to be developed for HIV control in mature epidemics | The Lancet |
| Hearst, Norman ; Chen, Sanny | 2004 | Condom Promotion for AIDS Prevention in the Developing World: Is It Working? | Multiple regions | Condoms are about 90 percent effective for preventing HIV transmission and HSV-2, and their use has grown rapidly in many countries. Condoms have produced substantial benefit in countries like Thailand, where both transmission and condom promotion are concentrated in the area of commercial sex. The public health benefit of condom promotion in settings with widespread heterosexual transmission, however, remains unestablished.In countries like Uganda that have curbed generalized epidemics, reducing the number of individuals' sex partners appears to have been more important than promoting the use of condoms | Studies in Family Planning |

| | | | | | |
|---|---|---|---|---|---|
| Feldblum, Paul J ; Kuyoh, Maureen A ; Bwayo, Job J ; Omari, Mohamed ; Wong, Emelita L ; Tweedy, Kathryn G ;Welsh, Michael J. | 2001 | Female condom introduction and sexually transmitted infection prevalence: results of a community intervention trial in Kenya | East Africa | female condom introduction did not enhance STI prevention at the sesites. It is unclear which aspects of the intervention | Health |
| Van Damme, Lut ; Ramjee, Gita ; Alary, Michel ; Vuylsteke, Bea ; Chandeying, Verapol ; Rees, Helen ; Sirivongrangson, Pachara ; Tshibaka, Léonard Mukenge ; Ettiègne-Traoré, Virginie ; Uaheowitchai, Charn ; Karim, Salim S. Abdool ; Mâsse, Benoît ; Perriëns, Jos ; Laga, Marie | 2002 | Effectiveness of COL-1492, a nonoxynol-9 vaginal gel, on HIV-1 transmission in female sex workers: a randomised controlled trial | Multiple regions | No significant effect of nonoxynol-9 on HSV-2 | The Lancet |
| Baeten, J. M ; Strick, L. B ; Lucchetti, A ; Whittington, W. L. H ; Sanchez, J ; Coombs, R. W ; Magaret, A ; Wald, A ; Corey, L ; Celum, C. | 2008 | Herpes simplex virus (HSV)-suppressive therapy decreases plasma and genital HIV-1 levels in HSV-2/HIV-1 coinfected women: a randomized, placebo-controlled, cross-over trial | South America | HIV-1 level was significantly lower during the valacyclovir arm, compared with the placebo arm ( 45% decrease [P<.001]), and cervical HIV-1 level (55% decrease [P<.001]), | Journal of Infectious Diseases |
| Fife KH, Warren TJ, Justus SE, Heitman CK | 2008 | An international, randomized, double-blind, placebo-controlled, study of valacyclovir for the suppression of herpes simplex virus type 2 genital herpes in newly diagnosed patients | Multiple regions | | Sexually Transmitted Diseases |

| | | | | | |
|---|---|---|---|---|---|
| Nagot N, Ouédraogo A, Foulongne V, Konaté I, Weiss HA, Vergne L | 2007 | Reduction of HIV-1 RNA levels with therapy to suppress herpes simplex virus | West Africa | | New England Journal of Medicine |
| Cowan, F. M ; Pascoe, S. J ; Barlow, K. L ; Langhaug, L. F ; Jaffar, S ; Hargrove, J. W ; Robinson, N. J ; Bassett, M. T ;Wilson, D ; Brown, D. W. G ; Hayes, R. J. | 2008 | A randomised placebo-controlled trial to explore the effect of suppressive therapy with acyclovir on genital shedding of HIV-1 and herpes simplex virus type 2 among Zimbabwean sex workers | Southren Africa | suppressive acyclovir therapy had no effect on the rate of HIV genital shedding despite a reduction in genital HSV-2 of 24% | Sexually Transmitted Infections |
| Mayaud, P ; LeGoff, J ; Weiss, H. A ; Grésenguet, G ; Nzambi, K ; Bouhlal, H ; Frost, E ; Pépin, J ; Malkin, J. E ; Hayes, R. J ; Mabey, D. C. W ; Bélec, L. | 2009 | Impact of acyclovir on genital and plasma HIV-1 RNA, genital herpes simplex virus type 2 DNA, and ulcer healing among HIV-1-infected African women with herpes ulcers: a randomized placebo-controlled trial | Multiple (Ghana, Central African Republic) | cervicovaginal HSV-2 DNA (RR, 0.69; 95% CI, 0.4-1.3), had a lower quantity of HSV-2 DNA (-0.99 log(10) copies/mL; 95% CI, -1.8 to -0.2 log(10) copies/mL), and were more likely to have a healed ulcer (RR, 1.26; 95% CI, 0.9-1.9). | Journal of Infectious Diseases |
| Dunne, E. F ; Whitehead, S ;. Sternberg, M ; Thepamnuay, S ; Leelawiwat, W ; McNicholl, J. M ; Sumanapun, S. Tappero, J. W ; Siriprapasiri, T ; Markowitz, L. | 2008 | Suppressive acyclovir therapy reduces HIV cervicovaginal shedding in HIV- and HSV-2-infected women, Chiang Rai, Thailand | East Asia | Acyclovir reduced HIV genital shedding and plasma viral load among HIV-1- and herpes simplex virus type 2-coinfected women | JAIDS, Journal of Acquired Immune Deficiency Syndromes |

| | | | | | |
|---|---|---|---|---|---|
| Tobian, A. A. R ; Serwadda, D ; Quinn, T. C ; Kigozi, G ; Gravitt, P. E ; Laeyendecker, O ;Charvat, B ;Ssempijja, V ; Riedesel, M ; Oliver, A. E ;Nowak, R. G ;Moulton, L. H ; Chen, M. Z ; Reynolds, S. J ; Wawer, M. J. ; Gray, R. H. | 2009 | Male Circumcision for the Prevention of HSV-2 and HPV Infections and Syphilis | east Africa | At 24 months, the cumulative probability of HSV-2 seroconversion was 7.8% in the intervention group and 10.3% in the control group (adjusted hazard ratio in the intervention group, 0.72; 95% confidence interval [CI], 0.56 to 0.92; P=0.008) | New England Journal of Medicine |
| Sobngwi-Tambekou, J ; Taljaard, D ; Lissouba, P ; Zarca, K ; Puren, A ; Lagarde, E ; Auvert, B. | 2009 | Effect of HSV-2 serostatus on acquisition of HIV by young men: results of a longitudinal study in Orange Farm, South Africa | Southren Africa | the protective effect of male circumcision on HIV acquisition appears independent of HSV-2 serostatus. | Journal of Infectious Diseases |
| Martin, Emily T ; Krantz, Elizabeth ; Gottlieb, Sami L ; Magaret, Amalia S ; Langenberg, Andria ; Stanberry, Lawrence ; Kamb, Mary ;Wald, Anna | 2009 | A Pooled Analysis of the Effect of Condoms in Preventing HSV-2 Acquisition | Multiple regions | Consistent condom users (used 100% of the time) had a 30% lower risk of HSV-2 acquisition compared with those who never used condoms (hazard ratio [HR], 0.70; 95% confidence interval [CI], 0.40-0.94) (P = .01). Risk for HSV-2 acquisition increased steadily and significantly with each unprotected sex act (HR, 1.16; 95% CI, 1.08-1.25) (P < .001). Condom effectiveness did not vary by gender. | Archives of Internal Medicine |

| | | | | | |
|---|---|---|---|---|---|
| Gottlieb SL, Giersing BK, Hickling J, Jones R, Deal C, Kaslow DC | 2019 | Meeting report: Initial World Health Organization consultation on herpes simplex virus (HSV) vaccine preferred product characteristics | Multiple regions | | Vaccine |
| Connie, ; Noah, ; Myron, | 2005 | Potential Effect of HIV Type 1 Antiretroviral and Herpes Simplex Virus Type 2 Antiviral Therapy on Transmission and Acquisition of HIV Type 1 Infection | Multiple regions | trials of ART or HSV-2 antiviral therapy are successful, prevention benefits may be difficult to translate to com-munity benefits; in general, trials of ART and HSV-2 antiviral therapy must be viewed as proof-of-concept studies. Favourable results can lead to the development of agents more appropriate to prevention and to operational strategies required for optimal implementation. | Oxford University Press (OUP) |
| Gregson, Simon ; Adamson, Saina ; Papaya, Spiwe ; Mundondo, Jephias ; Nyamukapa, Constance A ; Mason, Peter R ; Garnett, Geoffrey P ; Chandiwana, Stephen K ; Foster, Geoff ; Anderson, Roy M. | 2007 | Impact and Process Evaluation of Integrated Community and Clinic-Based HIV-1 Control: A Cluster-Randomised Trial in Eastern Zimbabwe | Southren Africa | Integrated peer education, condom distribution, and syndromic STI management did not reduce population-level HIV-1 and HSV-2 incidence in a declining epidemic, despite reducing HIV-1incidence in the immediate male target group. | PLoS Medicine |

| | | | | | |
|---|---|---|---|---|---|
| Paz-Bailey, G ; Ramaswamy, M ; Hawkes, S. J ; Geretti, A. M. | 2006 | Herpes simplex virus type 2: epidemiology and management options in developing countries | Multiple regions | multifaceted interventions are required to improve recognition of genital herpes, to prevent its spread and also to prevent its potential to promote HIV transmission in developing countriesare required to improve recognition of genital herpes, to prevent its spread and also to prevent its potential to promote HIV transmission in developing countries | Sexually Transmitted Infections |
| Jewkes, R ; Nduna, M ; Levin, J ; Jama, N ; Dunkle, K ; Puren, A ; Duvvury, N. | 2008 | Impact of Stepping Stones on incidence of HIV and HSV-2 and sexual behaviour in rural South Africa: cluster randomised controlled trial | Southren Africa | the programme was associated with a reduction of about 33%i n the incidence of HSV-2 (0.67, 0.46 to 0.97; P=0.036 | BMJ |
| Prichard, Mark N ; Kaiwar, Ravi ; Jackman, Winthrop T ; Quenelle, Debra C ; Collins, Deborah J ; Kern, Earl R ; Kemble, George M ; Spaete, Richard R. | 2005 | Evaluation of AD472, a live attenuated recombinant herpes simplex virus type 2 vaccine in guinea pigs | global | Immunization with AD472 at doses of 1 × 105 PFU generally precluded colonization of the ganglia or establishment of latency by the challenge virus. | Vaccine |
| Paz-Bailey, G ; Sternberg, M ; Puren, A. J ; Markowitz, L. E ; Ballard, R ; Delany, S ; Hawkes, S ; Nwanyanwu, O ; Ryan, C ; Lewis, D. A. | 2009 | improvement in healing and reduction in HIV shedding with episodic acyclovir therapy as part of syndromic management among men: a randomized, controlled trial | Southren Africa | Acyclovir improved ulcer healing--61% of those receiving acyclovir healed by day 7, compared with 42% of those receiving placebo (adjusted relative risk, 1.4 [95% confidence interval, 1.1-1.8]; P= .003) | Journal of Infectious Diseases |

| | | | | | |
|---|---|---|---|---|---|
| Baeten, J. M ; Reid, S. E ; Delany-Moretlwe, S ; Hughes, J. P ; Wang, R. S ; Wilcox, E ; Limbada, M ; Akpomiemie, G ; Corey, L ; Wald, A ;Celum, C. | 2012 | Clinical and virologic response to episodic acyclovir for genital ulcers among HIV-1 seronegative, herpes simplex virus type 2 seropositive African women: a randomized, placebo-controlled trial | Southren Africa | episodic acyclovir therapy resulted in modestly greater likelihood of lesion healing (HR=1.48, p=0.098; mean 5.1 vs. 6.0 days) and cessation of HSV shedding (HR=1.88, p=0.008; mean 3.0 vs. 5.0 days) compared with placebo, similar to results from high-income countries. | Sexually Transmitted Diseases |
| Celum, C ; Batten, J. M. | 2012 | Tenofovir-based pre-exposure prophylaxis for HIV prevention: evolving evidence | Global | Peri-coital use of 1% tenofovir gel in CAPRISA 004 reduced HIV-1acquisition by 39% and HSV-2 acquisition by 51%. Daily oral FTC/TDF demonstrated 44%reduction in HIV-1 acquisition among men who have sex with men (MSM) in iPrEx. Both studiesshowed higher efficacy among those with higher adherence. Efficacy of daily oral TDF and FTC/TDF was 66% and 73%, respectively, among HIV-1 uninfected partners in an HIV-1serodiscordant partnership in the Partners PrEP Study. Efficacy of daily oral FTC/TDF was 66%in young heterosexuals in Botswana in the TDF-2 trial. The FEM-PrEP and VOICE studies inAfrican women found no efficacy with oral FTC/TDF and TDF, respectively. Safety andtolerability were excellent and limited resistance was observed in seroconverters | Current Opinion in Infectious Diseases |

| | | | | | |
|---|---|---|---|---|---|
| Watson-Jones D, Weiss HA, Rusizoka M, Changalucha J, Baisley K, Mugeye K | 2008 | Effect of herpes simplex suppression on incidence of HIV among women in Tanzania | East Africa | RCT | New England Journal of Medicine |
| Fuchs, J ; Celum, C ; Wang, J ; Hughes, J ; Sanchez, J ; Cowan, F ; Reid, S ; Delany-Moretlwe, S; Corey, L; Wald, A. | 2010 | Clinical and virologic efficacy of herpes simplex virus type 2 suppression by acyclovir in a multi-continent clinical trial | Global | Overall, acyclovir was associated with a 47% [RR 0.53 (95% CI 0.46, 0.62)] reduction in GUD | Journal of Infectious Diseases |
| Moreno, R ; Nababan, H. Y ; Ota, E ; Wariki, W. M. V ; Ezoe, S ; Gilmour, S ; Shibuya, K. | 2014 | Structural and community-level interventions for increasing condom use to prevent the transmission of HIV and other sexually transmitted infections | Developing countires | no clear evidence that the intervention work | Cochrane Database of Systematic Reviews |

| | | | | | |
|---|---|---|---|---|---|
| Roxby, A. C.<br>Drake, A. L.<br>Ongecha-Owuor, F.<br>Kiarie, J. N.<br>Richardson, B; Matemo, D. N ; Overbaugh, J ; Emery, S ; John-Stewart, G. C ; Wald, A ; Farquhar, C. | 2012 | Effects of valacyclovir on markers of disease progression in postpartum women co-infected with HIV-1 and herpes simplex virus-2 | East Africa | Women randomized to valacyclovir suppressive therapy during pregnancy and postpartum had greater increases in CD4 counts and smaller increases in plasma HIV-1 RNA levels than women in the placebo arm. Valacyclovir suppression during pregnancy and breastfeeding may improve outcomes and delay antiretroviral therapy for HIV-1/HSV-2 co-infected women. | PLoS ONE |
| Reynolds, S. J ; Makumbi, F ; Newell, K ; Kiwanuka, N ; Ssebbowa, P ; Mondo, G ; Boaz, I ; Wawer, M. J ; Gray, R. H ; Serwadda, D ; Quinn, T. C. | 2012 | Effect of daily aciclovir on HIV disease progression in individuals in Rakai, Uganda, co-infected with HIV-1 and herpes simplex virus type 2: a randomised, double-blind placebo-controlled trial | east Africa | Aciclovir reduces the rate of disease progression, with the greatest effect in individuals with a high baseline viral load. Howevver, no significant difference in HIV disease progression existed between participants in the treatment group and those in the placebo group who had baseline HIV viral loads of less than 50,000 | Lancet Infectious Diseases |
| Phiri, S ; Hoffman, I. F ; Weiss, H. A ; Martinson, F ; Nyirenda, N ; Kamwendo, D ; Fiscus, S. A ; Chen, C. Y ; Miller, W. C ; Hoeven, L. van de ; Chilongozi, D ; Cohen, M. S ; Mayaud, P. | 2010 | Impact of aciclovir on ulcer healing, lesional, genital and plasma HIV-1 RNA among patients with genital ulcer disease in Malawi | Southren Africa | 85% of ulcers were healed at day 14 with no difference between treatment arms (risk ratio (RR)=1.02, 95% CI 0.93 to 1.11). Among 244 HIV-1/HSV-2 co-infected individuals, aciclovir reduced lesional HIV-1 RNA (adjusted RR=0.64, 95% CI 0.41 to 0.99) and seminal HIV-1 RNA (adjusted RR=0.59, 95% CI 0.40 to 0.88), but not cervical HIV-1 RNA or plasma HIV-1 RNA. | Sexually Transmitted Infections |

| | | | | | |
|---|---|---|---|---|---|
| Obiero, J ; Mwethera, P. G ;Wiysonge, C. S. | 2012 | Topical microbicides for prevention of sexually transmitted infections | Multiple regions | Tenofovir reduced the incidence of herpes simplex virus type 2 (HSV-2) infection (one trial, 426 women; RR 0.55; 95% CI 0.37 to 0.83) | Cochrane Database of Systematic Reviews |
| Lou, Yijun ; Qesmi, Redouane ; Wang, Qian ; Steben, Marc ; Wu, Jianhong ;Heffernan, Jane M. | 2012 | Epidemiological Impact of a Genital Herpes Type 2 Vaccine for Young Females | Global | vaccination of some females reduces infection in men, which then reduces infection in women. | PloS one |
| Lingappa, Jairam R ; Baeten, Jared M ; Wald, Anna ; Hughes, James P ;Thomas, Katherine K ;. Mujugira, Andrew; Mugo, Nelly ; Bukusi, Elizabeth A. | 2010 | Daily aciclovir for HIV-1 disease progression in people dually infected with HIV-1 and herpes simplex virus type 2: a randomised placebo-controlled trial | Multiple (East Africa, Southren Africa) | Acyclovir reduced risk of HIV-1 disease progression by 16%; 284 participants assigned acyclovir versus 324 assigned placebo reached the primary endpoint (hazard ratio [HR] 0.84, 95% CI 0.71-0.98, p=0.03). | Elsevier BV |
| Tanton, Clare ; Weiss, Helen A ; Rusizoka, Mary ; Legoff, Jerome ; Changalucha, John ; Baisley, Kathy ; Mugeye, Kokugonza ; Everett, Dean ; Belec, Laurent ; Clayton, Tim C. ; Ross, David A. ; Hayes, Richard J. ; Watson-Jones, Deborah | 2010 | Long-term impact of acyclovir suppressive therapy on genital and plasma HIV RNA in Tanzanian women: a randomized controlled trial | East Africa | there was little difference between acyclovir and placebo arms for cervico-vaginal HIV-1 RNA detection, Acyclovir 400mg bid is unlikely to be a useful long-term intervention to reduce HIV transmission. | The Journal of infectious diseases |
| Cowan, Frances M ; Pascoe, Sophie Js ; Langhaug, Lisa F ; Mavhu, Webster ; Chidiya, Samson ; Jaffar, Shabbar ; Mbizvo, Michael T ; Stephenson, Judith M ; Johnson, Anne M ; Power, Robert M ; Woelk, Godfrey ; Hayes, Richard J. | 2010 | The Regai Dzive Shiri project: results of a randomized trial of an HIV prevention intervention for youth | Southren Africa | no impact of the intervention on prevalence of HSV-2 | AIDS |
| Skoberne, M ; Cardin, R ; Lee, A ; Kazimirova, A ; Zielinski, V ; Garvie, D ; Lundberg, A ; Larson, S ; Bravo, F. J ; Bernstein, D. I ; Flechtner, J. B ; Long, D. | 2013 | An Adjuvanted Herpes Simplex Virus 2 Subunit Vaccine Elicits a T Cell Response in Mice and Is an Effective Therapeutic Vaccine in Guinea Pigs | Multiple regions | the frequency of recurrent viral shedding wasconsiderably reduced in GEN-003/MM-2-vaccinated animals but not in GEN-003- or MM-2-vaccinated animals | Journal of Virology |

| | | | | | |
|---|---|---|---|---|---|
| Morello, Christopher S ; Kraynyak, Kimberly A ; Levinson, Michael S ; hen, Zhijiang ; Lee, Kuo-Fen ; Spector, Deborah H. | 2012 | Inactivated HSV-2 in MPL/alum adjuvant provides nearly complete protection against genital infection and shedding following long term challenge and rechallenge | Multiple regions | optimized vaccine for-mulation of FI-HSV2 MPL/Alhydrogel given i.m. completely protected against detectable vaginal HSV-2 shedding in the majority of animal sand HSV-2 latent DNA in the DRG of all animals. | Elsevier BV |
| Free, C.; Roberts, I. G ; Abramsky, T ; Fitzgerald, M ; Wensley, F. | 2011 | A systematic review of randomised controlled trials of interventions promoting effective condom use | Multiple regions | The results are generally consistent withmodest benefits but there is considerable potential forbias due to poor trial quality. Because of the lowproportion of trials using the same outcome thepotential for bias from selective reporting of outcomesis considerable. Despite the public health importanceof increasing condom use there is little reliableevidence on the effectiveness of condom promotioninterventions | Journal of Epidemiology & Community Health |
| Qesmi, Redouane ; Wang, Qian ; Steben, Marc ; Wu, Jianhong ; Heffernan, Jane M. | 2012 | Epidemiological Impact of a Genital Herpes Type 2 Vaccine for Young Females | developing countires | Computer simulation shows that an adolescent-only vaccination program may be effective in eliminating HSV-2 disease, however, the success of extinction greatly depends on the level of vaccine uptake, the vaccine efficacy, the age of sexual maturity and safe sex practices. However, the time course of eradication would take many years | PloS one |

| | | | | | |
|---|---|---|---|---|---|
| Dropulic, Lesia K ; Cohen, Jeffrey I. | 2012 | The challenge of developing a herpes simplex virus 2 vaccine | developing countires | HSV-2 vaccines that reduce primary disease and shedding are an important goal for an HSV-2 vaccine. While sterilizing immunity would be ideal, the observation that persons can become infected with more than one strain of HSV-2 suggests that this might not be achievable. | Expert review of vaccines |
| Halford, William P ; Püschel, Ringo ; Gershburg, Edward ; Wilber, Andrew ; Gershburg, Svetlana ; Rakowski, Brandon | 2011 | A Live-Attenuated HSV-2 ICP0− Virus Elicits 10 to 100 Times Greater Protection against Genital Herpes than a Glycoprotein D Subunit Vaccine | Multiple regions | In vivoimaging demonstrated that a luciferase-expressing HSV-2 challenge virus failed toestablish a detectable infection in 0DNLS-immunized mice, whereas the same virus readily infected naı ̈ve and gD-2-immunized mice. Collectively, these results suggest that a HSV-2 vaccine might be more likely to prevent genital herpes if itcontained a live-attenuated HSV-2 virus rather than a single HSV-2 protein. | Plos one |
| Mujugira, Andrew ; Magaret, Amalia S ; Celum, Connie ; Baeten, Jared M ; Lingappa, Jairam R ; Morrow, Rhoda Ashley ; Fife, Kenneth H ; Delany-Moretlwe, Sinead ; de Bruyn, Guy ; Bukusi, Elizabeth A. ; Karita, Etienne ; Kapiga, Saidi ; Corey, Lawrence ; Wald, Anna; Partners in Prevention, H. S. V. H. I. V. Transmission Study Team | 2013 | Daily acyclovir to decrease herpes simplex virus type 2 (HSV-2) transmission from HSV-2/HIV-1 coinfected persons: a randomized controlled trial | East Africa, Southren Africa | Treatment of African HSV-2/HIV-1–infected persons with daily suppressive acyclovir did not decrease risk of HSV-2 transmission to susceptible partners | The Journal of infectious diseases |

| | | | | | |
|---|---|---|---|---|---|
| Doyle, Aoife M ; Ross, David A ; Maganja, Kaballa ; Baisley, Kathy ; Masesa, Clemen ;Andreasen, Aura ; Plummer, Mary L ; Obasi, Angela I. N ; Weiss, Helen A ; Kapiga, Said ;Watson-Jones, Debora ; Changalucha, John ; Hayes, Richard J. | 2010 | Long-Term Biological and Behavioural Impact of an Adolescent Sexual Health Intervention in Tanzania: Follow-up Survey of the Community-Based MEMA kwa Vijana Trial | East Africa | Prevalences ofthe primary outcomes HIV and HSV-2 were 1.8% and 25.9% in males and 4.0% and 41.4% in females, respectively. Theintervention did not significantly reduce risk of HIV (males adjusted prevalence ratio [aPR] 0.91, 95%CI 0.50–1.65; femalesaPR 1.07, 95%CI 0.68–1.67) or HSV-2 (males aPR 0.94, 95%CI 0.77–1.15; females aPR 0.96, 95%CI 0.87–1.06 | PLoS Medicine |
| Soares AR, Robaina MCS, Mendes GS, Silva TSL, Gestinari LMS, Pamplona OS | 2012 | Antiviral activity of extracts from Brazilian seaweeds against herpes simplex virus | Brazil | | Revista Brasileira de Farmacognosia |

| | | | | | |
|---|---|---|---|---|---|
| Alsallaq RA, Schiffer JT, Longini Jr IM, Wald A, Corey L, Abu-Raddad LJ | 2010 | Population level impact of an imperfect prophylactic HSV-2 vaccine | Developing countries | population and individual effects of HSV-2 vaccine | Sexually transmitted diseases |
| Johnston, Christine ; Koelle, David M ; Wald, Anna | 2014 | Current status and prospects for development of an HSV vaccine | global | the importance of the mucosal immune response, availability of novel adjuvants and delivery mechanisms, identification of T cell epitopes via proteomic screening and advancement in replication competent and replication-incompetent candidates. In addition, we have learned from past vaccine studies; we need to depend on objective evidence of seroconversion rather than the variable phenotype of clinical disease in preventative vaccine studies. Similarly, viral shedding should be measured rather than recurrence rate as the most sensitive tool to evaluate both prophylactic and therapeutic vaccines, to provide data on potential impact of vaccination on HSV transmission | Vaccine |

| | | | | | |
|---|---|---|---|---|---|
| Moussa HS, Ibrahem ABB, El-Sayed AFM, Mohammed FA | 2015 | In vitro evaluation of anti-microbial activities of marine Streptomyces against viral models, bacterial and fungal strains. | In vitro study aimed at developing countries | | International Journal of Virology |
| Roberts, Lindi; Liebenberg, Lenine; Barnabas, Shaun ; Passmore, Jo-Ann | 2012 | Vaginal microbicides to prevent human immunodeficiency virus infection in women: Perspectives on the female genital tract, sexual maturity and mucosal inflammation | Multiple regions | vaginal microbicides offer an important female-controlled strategy to prevent sexual transmission ofHIV. Use of antiretroviral drugs such as TFV 1% in coitally linked topical microbicide formulations hasrecently proven to be a moderately effective tool at preventing new infections in women 18 years and older | Best Practice & Research Clinical Obstetrics & Gynaecology |

| | | | | | |
|---|---|---|---|---|---|
| García, Patricia J ; Holmes, King K ; Cárcamo, César P ; Garnett, Geoff P ; Hughes, James P ; Campos, Pablo E ; Whittington, William L. H ; Peru, Preven Study Team | 2012 | Prevention of sexually transmitted infections in urban communities (Peru PREVEN): a multicomponent community-randomised controlled tria | South America | We report a non-significant reduction in prevalence of STIs in young adults, adjusted for baseline prevalence, in intervention cities compared with control cities (relative risk 0·84, 95% CI 0·69–1·02; p=0·096) | Lancet (London, England) |
| Hallfors, D. D ; Cho, H. S ; Rusakaniko, S ; Mapfumo, J ; Iritani, B ; Zhang, L ; Luseno, W ; Miller, T. | 2015 | The impact of school subsidies on HIV-related outcomes among adolescent female orphans | Southren Africa | The two groups did not differ on HIV or HSV-2 biomarkers | Journal of Adolescent Health |
| Low, A. J ; Nagot, N ; Weiss, H. A ; Konate, I ; Kania, D ; Segondy, M ; Meda, N ; Perre, P. van de ; Mayauda, P. | 2016 | Herpes Simplex Virus Type-2 Cervicovaginal Shedding Among Women Living With HIV-1 and Receiving Antiretroviral Therapy in Burkina Faso: An 8-Year Longitudinal Study | West Africa | Cervicovaginal HSV-2 DNA was detected in 42% of women (99 of 236) in 8.2% of visits . ART was associated with a reduction in the odds of HSV-2 shedding, which declined for each year of ART use (odds ratio [OR], 0.74; 95% confidence interval [CI], .59–.92). | Journal of Infectious Diseases |

| | | | | | |
|---|---|---|---|---|---|
| Yuan, Tanwei ; Fitzpatrick, Thomas ; Ko, Nai-Ying ; Cai, Yong ; Chen, Yingqing ; Zhao, Jin ; Li, Linghua ; Xu, Junjie ; Gu, Jing ; Li, Jinghua ; Hao, Chun ; Yang, Zhengrong ; Cai, Weiping ; Cheng, Chien-Yu ;Luo,;Zhenzhou Zhang, Kechun Wu, Guohui Meng, Xiaojun Grulich, Andrew E. Hao, Yuantao Zou, Huachun Majiwa, M. Onyango, C. O. Kuile, F. O. ter Phillips-Howard, P. A. | 2019 | Circumcision to prevent HIV and other sexually transmitted infections in men who have sex with men: a systematic review and meta-analysis of global data | Developing countires | Circumcision was associated with reduced odds of herpes simplex virus (HSV) infection among MSM overall (0·84, 0·75–0·95; k=5; I²=0%) | The Lancet. Global health |
| Stoner MCD, Neilands TB, Kahn K, Hughes JP, Gómez-Olivé FX, Twine R | 2019 | Multilevel measures of education and pathways to incident Herpes simplex virus type 2 in adolescent girls and young women in South Africa | Southern Africa | | Journal of Adolescent Health |

| | | | | | |
|---|---|---|---|---|---|
| Farr Zuend, Christina , Nomellini, John F , Smit, John, Horwitz, Marc S , Townsend, J. | 2018 | Generation of a Dual-Target, Safe, Inexpensive Microbicide that Protects Against HIV-1 and HSV-2 Disease | Southren Africa | Protection from HSV-2 disease was strongly associated with early cytokine production in the vaginal tract. Our data support the potential for a dual-target microbicide that can protect against both HIV-1 and HSV-2, which could have an enormous impact on public health | Scientific Reports |
| Johnston, Christine ; Gottlieb, Sami L ; Wald, Anna | 2016 | Status of vaccine research and development of vaccines for herpes simplex virus | Multiple regions | provide an overview of efforts to develop HSV-2 vaccines, including a discussion of the clinical need for an HSV vaccine, and status of research and development with an emphasis on recent insights from trials of vaccine candidates in clinical testing. In addition, we will touch upon aspects of HSV vaccine development relevant to low and middle income countries | Vaccine |

| | | | | | |
|---|---|---|---|---|---|
| Jiang, Yu-Chen ; Feng, Hui ; Lin, Yu-Chun ; Guo, Xiu-Rong | 2016 | New strategies against drug resistance to herpes simplex virus | Multiple regions | new antiviral drugs with different antiviral actions, includ- ing new antiviral targets, new antiviral mechanisms, and new antiviral molecules, are required. Janus-type nucleosides have two different faces (mimicking the natural purine and pyrimidine systems) in one molecule, and these drugs may pair with diverse natural bases via rotation around the glycosyl bond, which further induces viral lethal mutation. Therefore, unique Janus-type nucleoside analogues possess great potential in the exploitation of new lethal mutagenesis drugs as novel strategies for antiviral chemotherapy. | International journal of oral science |
| Shahzad, Naveed ; Farooq, Roman ; Aslam, Bilal ; Umer, Muhammad | 2017 | Microbicides for the Prevention of HPV, HIV-1, and HSV-2: Sexually Transmitted Viral Infections | South Asia | More trials can guide for more development of more rationally, accurately, behaviorally, and socially accepted and single solution maximum potential STIs. | Fundamentals of Sexually Transmitted Infections (Book) |

| | | | | | |
|---|---|---|---|---|---|
| Egan, Kevin P ; Hook, Lauren M ; Naughton, Alexis ; Pardi, Norbert ; Awasthi, Sita ; Cohen, Gary H ; Weissman, Drew ; Friedman, Harvey M. | 2020 | An HSV-2 nucleoside-modified mRNA genital herpes vaccine containing glycoproteins gC, gD, and gE protects mice against HSV-1 genital lesions and latent infection | Global | In the two experiments, them RNA vaccine prevented death and genital disease in 54/54(100%) mice infected with HSV-1 and 20/20 (100%) with HSV-2. They prevented HSV DNA from reaching the doral root ganglia,the site of virusl atency, in 29/30(97%) mice infected with HSV-1 and 10/10 (100%) with HSV-2. | PLOS Pathogens |
| Magaret, Amalia S ; Mujugira, Andrew ; Hughes, James P ; Lingappa, Jairam ; Bukusi, Elizabeth A ; DeBruyn, Guy ;Delany-Moretlwe, Sinead ; Fife, Kenneth H ; Gray, Glenda E ; Kapiga, Saidi ; Karita, Etienne ; Mugo, Nelly R ; Rees, Helen ; Ronald, Allan ; Vwalika, Bellington ; Were, Edwin ; Celum, Connie ; Wald, Anna ; Partners in Prevention, H. S. V. H. I. V. Transmission Study Team | 2016 | Effect of Condom Use on Per-act HSV-2 Transmission Risk in HIV-1, HSV-2-discordant Couples | Multiple regions | Transmission of HSV-2 occurred in 68 couples, including 17 with susceptible women and 51 with susceptible men. The highest rate of transmission was from men to women: 28.5 transmissions per 1000 unprotected sex acts. We found that condoms were differentially protective against HSV-2 transmission by sex; condom use reduced per-act risk of transmission from men to women by 96% (P < .001) and marginally from women to men by 65% (P = .060). | Clinical infectious diseases : an official publication of the Infectious Diseases Society of America |
| Ceña-Diez, Rafael; Vacas-Córdoba, Enrique ; García-Broncano, Pilar ; de la Mata, F. J. ; Gómez, Rafael ; Maly, Marek ; Muñoz-Fernández, M. Ángeles | 2016 | Prevention of vaginal and rectal herpes simplex virus type 2 transmission in mice: mechanism of antiviral action | Multiple regions | Topical vaginal or rectal administration of G1-S4 or G2-S16 prevents HSV-2 transmission in BALB/c mice in values close to 100% | International journal of nanomedicine |

HSV-2 and HIV interactions

| **Authors** | **Year** | **Title** | **Geographical area of study population** | **Study design** | **sample size** | **Journal** | **Study topic** | **Main findings** |
|---|---|---|---|---|---|---|---|---|
| Mbopi-Kéou, F. X.,Grésenguet, G.,Mayaud, P.,Weiss, H. A.,Gopal, R.,Matta, M.,Paul, J. L.,Brown, D. W. G.,Hayes, R. J.,Mabey, D. C. W.,Bélec, L. | 2000 | Interactions between herpes simplex virus type 2 and human immunodeficiency virus type 1 infection in African women: opportunities for intervention | central Africa (CAR) | cross sectional study | 300 | Journal of Infectious Diseases | biological relationship: comparing serology, genital DNA and **shedding** in HIV infected and non infected | HSV shedding, antibody presence and HSV DNA presence were all more prevalent in HIV positive persons. Positive significant correlation between HIV RNA and HSV-2 DNA levels in genitals in dually infected women. They suggested more RCT research with HIV shedding or incidence as outcomes |
| Weiss, H. A.,Buvé, A.,Robinson, N. J.,Dyck, E. van,Kahindo, M.,Anagonou, S.,Musonda, R.,Zekeng, L.,Morison, L.,Caraël, M.,Laga, M.,Hayes, R. J. | 2001 | The epidemiology of HSV-2 infection and its association with HIV infection in four urban African populations | West, East and Southern Africa | cross sectional study | 2000 | AIDS | epidemiological /prevalence relationship | strong association between HSV-2 and Hiv prevalence (aOR between 4 and 8 in different groups) |

| | | | | | | | | |
|---|---|---|---|---|---|---|---|---|
| Auvert, B.,Ballard, R.,Campbell, C.,Caraël, M.,Carton, M.,Fehler, G.,Gouws, E.,MacPhail, C.,Taljaard, D.,Dam, J. van,Williams, B. | 2001 | HIV infection among youth in a South African mining town is associated with herpes simplex virus-2 seropositivity and sexual behaviour | Southern Africa | cross sectional study | 1507 | AIDS | epidemiological /prevalence relationship | HSV-2 a strong independent RF for HIV (OR 5.3 for men and 8.4 for women). Hsv plays major role in spread of HIV. Suggest prompt treatment of STIs and encourage condom use and school based sexual education |
| Gray RH, Wawer MJ, Brookmeyer R, Sewankambo NK, Serwadda D, Wabwire-Mangen F | 2001 | Probability of HIV-1 transmission per coital act in monogamous, heterosexual, HIV-1-discordant couples in Rakai, Uganda | East Africa | | | The Lancet | | |

| | | | | | | | | |
|---|---|---|---|---|---|---|---|---|
| McClelland, R. S.,Wang, C. C.,Overbaugh, J.,Richardson, B. A.,Corey, L.,Ashley, R. L.,Mandaliya, K.,Ndinya-Achola, J.,Bwayo, J. J.,Kreiss, J. K. | 2002 | Association between cervical shedding of herpes simplex virus and HIV-1 | East Africa | cross sectional study | 200 | AIDS | Biological: relationship between cervical shedding | significant association between HIV RNA and HSV DNA cervical shedding (r=0.24 Pearson). Researchers suggest more prospective studies to investigate relationship between non ulcerative HSV reactivation and hIV shedding |
| Mbizvo, E. M.,Sia, E. M.,Stray-Pedersen, B.,Chirenje, M. Z.,Munjoma, M.,Hussain, A. | 2002 | Association of herpes simplex virus type 2 with the human immunodeficiency virus among urban women in Zimbabwe | Southern Africa | cross sectional study | 393 | International Journal of STD & AIDS | epidemiological /prevalence relationship | HSV-2 positive women were twice as likely to be HIV positive (OR 2.05). Hsv-1 thought to contribute more to HIV than syphilis. Authors communicate urgent need for HSV 2 vaccine |

| | | | | | | | | |
|---|---|---|---|---|---|---|---|---|
| Rodríguez, M. del M. P.,Obasi, A.,Mosha, F.,Todd, J.,Brown, D.,Changalucha, J.,Mabey, D.,Ross, D.,Grosskurth, H.,Hayes, R. | 2002 | Herpes simplex virus type 2 infection increases HIV incidence: a prospective study in rural Tanzania | East Africa | unmatched case control study nested within a prospective RCT | 763 | AIDS | epidemiological: prevalent HSV and incident HIV | significant association between HSV and HIV incidence. Stronger in men (OR 6.2) than women(OR 1.32). Stronger association in those who seroconverted during study (new HSV infections). Population attributable fraction of incident HIV due to HSV was 74% in en and 22% in women. HSv plays big role in HIV transmission in Tanzania and urgent need for HSV control measures to control HIV in Africa. |
| Ahmed, H. J.,Mbwana, J.,Gunnarsson, E.,Ahlman, K.,Guerino, C.,Svensson, L. A.,Mhalu, F.,Lagergard, T. | 2003 | Etiology of genital ulcer disease and association with human immunodeficiency virus infection in two Tanzanian cities | East Africa | cross sectional study | 195 | Sexually Transmitted Diseases | epidemiological /prevalence relationship | HIV prevalence significant higher in HSV positive people than negative in all areas. Women with HSV more likely to be HIV positive than men with HSV |

| | | | | | | | | |
|---|---|---|---|---|---|---|---|---|
| Mbopi-Kéou, F. X.,Legoff, J.,Grésenguet, G.,Ali, Si-Mohamed,Matta, M.,Mayaud, P.,Andreoletti, L.,Malkin, J. E.,Weiss, H.,Brown, D.,Bélec, L. | 2003 | Genital shedding of herpes simplex virus-2 DNA and HIV-1 RNA and proviral DNA in HIV-1- and herpes simplex virus-2-coinfected African women | central Africa (CAR) | cross sectional study | 300 | JAIDS, Journal of Acquired Immune Deficiency Syndromes | biological: HIV pro virla DNA genital load in dually infected persons (effect of HSV on genital shedding)/transmission | those infected with serum HSV and HIV were twice as likely to be shedding HSV than those HSV positive and HIV negative. No association between genital proviral HIV DNA and genital HSV DNA levels, or between genital HIV RNA and genital HSV DNA shedding. There was a significant correlation between genital HIV RNA and genital HSV dNA amongst dually infected women, but only in HSV shedders. |

| | | | | | | | | |
|---|---|---|---|---|---|---|---|---|
| Serwadda, D.,Gray, R. H.,Sewankambo, N. K.,Wabwire-Mangen, F.,Chen, M. Z.,Quinn, T. C.,Lutalo, T.,Kiwanuka, N.,Kigozi, G.,Nalugoda, F.,Meehan, M. P.,Morrow, R. A.,Wawer, M. J. | 2003 | Human immunodeficiency virus acquisition associated with genital ulcer disease and herpes simplex virus type 2 infection: a nested case-control study in Rakai, Uganda | East Africa | case control study | 248 | Journal of Infectious Diseases | biological: timing of HSV relative to HIV seroconversion | those who seroconverted (HIV) were more likely to be HSV seropositive (OR 1.7), HIV viral load was higher in dually infected |
| Reynolds, S. J.,Risbud, A. R.,Shepherd, M. E.,Zenilman, J. M.,Brookmeyer, R. S.,Paranjape, R. S.,Divekar, A. D.,Gangakhedkar, R. R.,Ghate, M. V.,Bollinger, R. C.,Mehendale, S. M. | 2003 | Recent herpes simplex virus type 2 infection and the risk of human immunodeficiency virus type 1 acquisition in India | South Asia | cohort study | 2737 | Journal of Infectious Diseases | effect of genital herpes (new and old infections) on HIV acquisition | HSV prevalent cases more likely to acquire HIV (hazard ratio 1.67). Stronger relationship between recent incident HSV (<6m) and HIV acquisition (hazard ratio 3.81). Suggest that HSV prevention will help reduce HIV acquisition risk |

| | | | | | | | | |
|---|---|---|---|---|---|---|---|---|
| Baeten, J. M.,McClelland, R. S.,Corey, L.,Overbaugh, J.,Lavreys, L.,Richardson, B. A.,Wald, A.,Mandaliya, K.,Bwayo, J. J.,Kreiss, J. K. | 2004 | Vitamin A supplementation and genital shedding of herpes simplex virus among HIV-1-infected women: a randomized clinical trial | East Africa | RCT | 376 | Journal of Infectious Diseases | effect of vitamin A on HSV shedding in dually infected people | vitamin A unlikely to reduce vaginal HSV 2 shedding in dually infected, secondary finding was that those who were HSV shedding had higher HIV shedding |
| Duffus, W. A.,Mermin, J.,Bunnell, R.,Byers, R. H.,Odongo, G.,Ekwaru, P.,Downing, R. | 2005 | Chronic herpes simplex virus type-2 infection and HIV viral load | East Africa | cross sectional study | 339 | International journal of STD & AIDS | natural history of HSV and HIV coinfection: effect of HSV on viral load and cd4 | coinfection associated with higher HIV viral load. Chronic HSV may have a negative impact on clinical course of HIV |
| Nadal, S. R.,Calore, E. E.,Manzione, C. R.,Horta, S. C.,Ferreira, A. F.,Almeida, L. V. | 2005 | Hypertrophic herpes simplex simulating anal neoplAsia in AIDS patients: report of five cases | South America | case series | 5 | Diseases of the Colon and Rectum | effect of HSV on HIV clicical course/natural hx: anal neoplAsia | HSV 2 may cause verrucous lesion that can lead to neolpAsia in AIDS pts on HAART |

| | | | | | | | | |
|---|---|---|---|---|---|---|---|---|
| Gita, Ramjee,Williams, B.,Gouws, E.,Dyck, E. van,Deken, B. de,Karim, S. A. | 2005 | The impact of incident and prevalent herpes simplex virus-2 infection on the incidence of HIV-1 infection among commercial sex workers in South Africa | Southern Africa | cohort study | 187 | JAIDS, Journal of Acquired Immune Deficiency Syndromes | effect of incident and prevalent HSV on HIV acquisition | no sig difference in HIV incidence between people who were HSV positive or negative throughout trial. However, those with incident HSV were more likely to acquire HIV than those with prevalent HSV (hazard ratio 6). So, suggests that new HSV infection increases HIV acquisition risk and the risk decreases with time, this effect seems greater in men. |

| | | | | | | | | |
|---|---|---|---|---|---|---|---|---|
| Chu, K.,Jiamton, S.,Pepin, J.,Cowan, F.,Mahakkanukrauh, B.,Suttent, R.,Robinson, N. J.,Deslandes, S.,Frost, E.,Chaisilwattana, P.,Suthipinittharm, P.,Grosskurth, H.,Brown, D.,Jaffar, S. | 2006 | Association between HSV-2 and HIV-1 viral load in semen, cervico-vaginal secretions and genital ulcers of Thai men and women | East Asia | cross sectional study | 104 | International Journal of STD & AIDS | effect of HSV on HIV viral load in different samples | presence of HSV shedding did not affect HIV viral load in semen and vagina. Findings suggest that HSV does not cause increased viral loads in genital secretions. |
| Cowan, F. F.,Pascoe, S. J. S.,Barlow, K. L.,Langhaug, L. F.,Jaffar, S.,Hargrove, J. W.,Robinson, N. J.,Latif, A. S.,Bassett, M. T.,Wilson, D.,Brown, D. W. G.,Hayes, R. J. | 2006 | Association of genital shedding of herpes simplex virus type 2 and HIV-1 among sex workers in rural Zimbabwe | Southern Africa | cross sectional study | 214 | AIDS | Shedding/transmission | almost all HIV positive were co infected (95%). No apparent relationship between HSV shedding and HIV 1 shedding |

| | | | | | | | | |
|---|---|---|---|---|---|---|---|---|
| Lama, J. R.,Lucchetti, A.,Suarez, L.,Laguna-Torres, V. A.,Guanira, J. V.,Pun, M.,Montano, S. M.,Celum, C. L.,Carr, J. K.,Sanchez, J.,Bautista, C. T.,Sanchez, J. L. | 2006 | Association of herpes simplex virus type 2 infection and syphilis with human immunodeficiency virus infection among men who have sex with men in Peru | South America | cross sectional study | 3280 | Journal of Infectious Diseases | prevalence | 80% of HIV seroositive were HSV2 seropositive. Hsv2 seropositivity a significant predictor of HIV seropositiviy. Hsv control measure should help reduce HIV infection in msmin Peru |
| Ouedraogo, A.,Nagot, N.,Vergne, L.,Konate, I.,Weiss, H. A.,Defer, M. C.,Foulongne, V.,Sanon, A.,Andonaba, J. B.,Segondy, M.,Mayaud, P.,Perre, P. D. van | 2006 | Impact of suppressive herpes therapy on genital HIV-1 RNA among women taking antiretroviral therapy: a randomized controlled trial | West Africa | RCT | 60 | AIDS | effect of HSV on genital HIV rna SHEDDING | overall no association between valaciclovir use and HIV shedding. But amongst the HIV sheddersonly , valacyclovir was associated with reduce genital HIV shedding. Conc: HSV-2 may cause replication of genital HIV in dually infected women on haart |

| | | | | | | | | |
|---|---|---|---|---|---|---|---|---|
| Ao TTH, Sam NE, Masenga EJ, Seage GR, III, Kapiga SH | 2006 | Human immunodeficiency virus type 1 among bar and hotel workers in northern Tanzania: the role of alcohol, sexual behavior, and herpes simplex virus type 2 | East Africa | | | Sexually Transmitted Diseases. | | |

| | | | | | | | | |
|---|---|---|---|---|---|---|---|---|
| Legoff, Jérôme,Bouhlal, Hicham,Grésenguet, Gérard,Weiss, Helen,Khonde, Nzambi,Hocini, Hakim,Désiré, Nathalie,Si-Mohamed, Ali,de Dieu Longo, Jean,Chemin, Cécile,Frost, Eric,Pépin, Jacques,Malkin, Jean-Elie,Mayaud, Philippe,Bélec, Laurent | 2006 | Real-time PCR quantification of genital shedding of herpes simplex virus (HSV) and human immunodeficiency virus (HIV) in women coinfected with HSV and HIV | West and central Africa | cross sectional study | 269 | Journal of clinical microbiology | accuracy of real time pcr in detecting HSV and HIV shedding in dually infected persons | real time PCR is a suitable method of measuring HIV and HSV shedding for future studies |
| Santos, F. C.,Oliveira, S. A. de,Setúbal, S.,Camacho, L. A. B.,Faillace, T.,Leite, J. P. G.,Velarde, L. G. C. | 2006 | Seroepidemiological study of herpes simplex virus type 2 in patients with the acquired immunodeficiency syndrome in the City of Niterói, Rio de Janeiro, Brazil | South America | cross sectional study | 150 | Memórias do Instituto Oswaldo Cruz | associations in prevalence | 52% of participant were HSV seropositive. Only 20% of HSV positive were aware of their infection. |

| | | | | | | | | |
|---|---|---|---|---|---|---|---|---|
| LeGoff, J.,Weiss, H. A.,Gresenguet, G.,Nzambi, K.,Frost, E.,Hayes, R. J.,Mabey, D. C. W.,Malkin, J. E.,Mayaud, P.,Belec, L. | 2007 | Cervicovaginal HIV-1 and herpes simplex virus type 2 shedding during genital ulcer disease episodes | West and central Africa - urban | cross sectional study | 441 | AIDS | correlates of HSV and HIV SHEDDING | genital herpes infection was associated with increased genital HIV shedding and plasma HIV viral load in co infected women with genital ulcer |
| Ziyaeyan, M.,Alborzi, A.,Japoni, A.,Kadivar, M.,Davarpanah, M. A.,Pourabbas, B.,Abassian, A. | 2007 | Frequency of acyclovir-resistant herpes simplex viruses isolated from the general immunocompetent population and patients with acquired immunodeficiency syndrome | South Asia | cross sectional study | 133 | International Journal of Dermatolog y | occurance of acyclovir resistance HSV strains in AIDS patients | AIDS patients are much more likely to be infected with acyclovir resistant strains of HSV2. alternative anti virals should be used in AIDS pts |
| Zuckerman, R. A.,Lucchetti, A.,Whittington, W. L. H.,Sánchez, J.,Coombs, R. W.,Zuñiga, R.,Magaret, A. S.,Wald, A.,Corey, L.,Celum, C. | 2007 | Herpes simplex virus (HSV) suppression with valacyclovir reduces rectal and blood plasma HIV-1 levels in HIV-1/HSV-2-seropositive men: a randomized, double-blind, placebo-controlled crossover trial | South America | cross over RCT | 20 | Journal of Infectious Diseases | effect of valcyclovir on genital and serum HIV levels | valacyclovir was associated with a significant reduction in rectal and plasma HIV levels in co-infected msm not on ART. Valacyclovir may be useful in affecting the clinical course of HIV in coinfected men not on ART and also may reduce transmission. |

| | | | | | | | | |
|---|---|---|---|---|---|---|---|---|
| Drake, Alison L.,John-Stewart, Grace C.,Wald, Anna,Mbori-Ngacha, Dorothy A.,Bosire, Rose,Wamalwa, Dalton C.,Lohman-Payne, Barbara L.,Ashley-Morrow, Rhoda,Corey, Lawrence,Farquhar, Carey | 2007 | Herpes simplex virus type 2 and risk of intrapartum human immunodeficiency virus transmission | East Africa | nested case control study | 175 | Obstetrics and gynecology | transmission: effect of HSV2 on HIV transmission during birth | 87% of the pregnant mothers with HIV were dually had HSV. |
| Baeten, J. M.,Benki, S.,Chohan, V.,Lavreys, L.,McClelland, R. S.,Mandaliya, K.,Ndinya-Achola, J. O.,Jaoko, W.,Overbaugh, J. | 2007 | Hormonal contraceptive use, herpes simplex virus infection, and risk of HIV-1 acquisition among Kenyan women | East Africa | cohort study | 1206 | AIDS | effect of genital herpes infection on the relationship between hormonal contraceptive use and HIV ACQUISITION | both hormonal contraceptives and HSV 2 infection were separately associated with acquisition, however HSV infection did not increase the HIV risk in hormone contraceptive users. They both increased the risk separately. |

| | | | | | | | | |
|---|---|---|---|---|---|---|---|---|
| Brown, J. M.,Wald, A.,Hubbard, A.,Rungruengthanakit, K.,Chipato, T.,Rugpao, S.,Mmiro, F.,Celentano, D. D.,Salata, R. S.,Morrison, C. S.,Richardson, B. A.,Padian, N. S. | 2007 | Incident and prevalent herpes simplex virus type 2 infection increases risk of HIV acquisition among women in Uganda and Zimbabwe | East and Southern Africa | prospective cohort study | 4531 | AIDS | effect of incident and prevalent HSV on HIV acquisition | prevalent and incident HSV associated with higher risk of HIV acquisition in both countries. Incident HSV had more of an effect (hazarad ratio 4.6 to 8.6). Hsv had a greater effect in zimbabwe. POPULATION ATTRIBUTABLE RISK for HIV due to incidence and prevalent HSV was 42% in India and 65% in zimbabwe. HSV PLAYS BIG ROLE IN HIV ACQUISITION. |

| | | | | | | | | |
|---|---|---|---|---|---|---|---|---|
| Rebbapragada, Anuradha,Wachihi, Charles,Pettengell, Christopher,Sunderji, Sherzana,Huibner, Sanja,Jaoko, Walter,Ball, Blake,Fowke, Keith,Mazzulli, Tony,Plummer, Francis A.,Kaul, Rupert | 2007 | Negative mucosal synergy between Herpes simplex type 2 and HIV in the female genital tract | East Africa | cross sectional study | 102 | AIDS (London, England) | mucosal interactions between HSV and HIV in the genital tract and effect on transmission | found 'negative mucosal synergy' where there is a vicious cycle. Genital HSV causes increased HIV target cells- HIV infection affects genital HSV immune regulation- HSV reactivation causes increased transmission of HSV and HIV. |
| Freeman, E. E.,Orroth, K. K.,White, R. G.,Glynn, J. R.,Bakker, R.,Boily, M. C.,Habbema, D.,Buvé, A.,Hayes, R. | 2007 | Proportion of new HIV infections attributable to herpes simplex 2 increases over time: simulations of the changing role of sexually transmitted infections in sub-Saharan African HIV epidemics | West, East and Southern Africa | cross sectional modelling study | 999 | Sexually Transmitted Infections | modelling effect of HSV on HIV incidence (Population attributable fraction) | HSV2 has an increasing effect on HIV incidence as a model epidemic progresses (PAF 0f 35-48% 15 years in to epidemic). Effect of HSV on HIV infectivity (transmission) thought to have more of an effect than effect of HSV on HIV acquisition. Hsv2 programmes may be essential in HIV epidemics. |

| | | | | | | | | |
|---|---|---|---|---|---|---|---|---|
| Nagot, N.,Ouédraogo, A.,Foulongne, V.,Konaté, I.,Weiss, H. A.,Vergne, L.,Defer, M. C.,Djagbaré, D.,Sanon, A.,Andonaba, J. B.,Becquart, P.,Segondy, M.,Vallo, R.,Sawadogo, A.,Perre, P. van de,Mayaud, P. | 2007 | Reduction of HIV-1 RNA levels with therapy to suppress herpes simplex virus | West aFrica | RCT | 136 | New England Journal of Medicine | HIV shedding/trans mission: effect of valcyclovir | valcyclovir significantly reduced genital and plasma HIV rna level in dually infected women not on HAART. May be useful in HIV control. |

| | | | | | | | | |
|---|---|---|---|---|---|---|---|---|
| Kapiga, S. H.,Sam, N. E.,Bang, H. J.,Ni, Q. H.,Ao, T. T. H.,Kiwelu, I.,Chiduo, S.,Ndibe, U.,Seage, G., III,Coplan, P.,Shao, J.,Rosenberg, Z. F.,Essex, M. | 2007 | The role of herpes simplex virus type 2 and other genital infections in the acquisition of HIV-1 among high-risk women in Northern Tanzania | East Africa | cohort study | 845 | | effect of HSV on HIV ACQUISITION | risk of HIV acquisition was increased in those with prevalent genital HSV (hazarad ratio 4.3) and even more elevated in incident HSV (HR .55). Hsv2 is a big part of HIV acquisition |
| Celum, C.,Wald, A.,Hughes, J.,Sanchez, J.,Reid, S.,Delany-Moretlwe, S.,Cowan, F.,Casapia, M.,Ortiz, A.,Fuchs, J.,Buchbinder, S.,Koblin, B.,Zwerski, S.,Rose, S.,Wang, J.,Corey, L. | 2008 | Effect of aciclovir on HIV-1 acquisition in herpes simplex virus 2 seropositive women and men who have sex with men: a randomised, double-blind, placebo-controlled trial | South America Southern Africa | RCT | 3172 | | HIV ACQUISITION: effect of acyclovir | Acyclovir did NOT reduce HIV acquisition in HSV seropositive MSM and women |

| | | | | | | | | |
|---|---|---|---|---|---|---|---|---|
| Dunne, E. F ; Whitehead, S ;. Sternberg, M ; Thepamnuay, S ; Leelawiwat, W ; McNicholl, J. M ; Sumanapun, S. Tappero, J. W ; Siriprapasiri, T ; Markowitz, L. | 2008 | Suppressive acyclovir therapy reduces HIV cervicovaginal shedding in HIV- and HSV-2-infected women, Chiang Rai, Thailand | East Asia | | | JAIDS, Journal of Acquired Immune Deficiency Syndromes | | Acyclovir reduced HIV genital shedding and plasma viral load among HIV-1- and herpes simplex virus type 2-coinfected women |

| | | | | | | | | |
|---|---|---|---|---|---|---|---|---|
| Watson-Jones, D.,Weiss, H. A.,Rusizoka, M.,Changalucha, J.,Baisley, K.,Mugeye, K.,Tanton, C.,Ross, D.,Everett, D.,Clayton, T.,Balira, R.,Knight, L.,Hambleton, I.,Goff, J. le,Belec, L.,Hayes, R. | 2008 | Effect of herpes simplex suppression on incidence of HIV among women in Tanzania | East Africa | RCT | 821 | New England Journal of Medicine | effect of HSV suppression on HIV acquisition (acyclovir) | acyclovir had NO effect on hi acquisition in tanzanian women with HSV |
| Mayaud, P.,Nagot, N.,Konaté, I.,Ouedraogo, A.,Weiss, H. A.,Foulongne, V.,Defer, M. C.,Sawadogo, A.,Segondy, M.,Perre, P. van de | 2008 | Effect of HIV-1 and antiretroviral therapy on herpes simplex virus type 2: a prospective study in African women | West Africa | cohort study | 120 | Sexually Transmitted Infections | effect of HIV and haart on HSV natural hx | those with HIV had more frequent HSV recurrence. Haart did not significantly help. Hsv therapy still recommended for those with HSV and on haart. |

| | | | | | | | | |
|---|---|---|---|---|---|---|---|---|
| Abu-Raddad, Laith J.,Magaret, Amalia S.,Celum, Connie,Wald, Anna,Longini, Ira M., Jr.,Self, Steven G.,Corey, Lawrence | 2008 | Genital herpes has played a more important role than any other sexually transmitted infection in driving HIV prevalence in Africa | East and West Africa | modelling study | 999 | PloS one | contribution of HSV to HIV (PAF as an outcome) | HSV2 has had a huge role in HIV epidemics in SSA. PAF up tp 35% in general populations in Africa. Hsv has facilitated spread of HIV through low risk groups. This supports need for a HSV 2 vaccine |
| Baeten, J. M.,Strick, L. B.,Lucchetti, A.,Whittington, W. L. H.,Sanchez, J.,Coombs, R. W.,Magaret, A.,Wald, A.,Corey, L.,Celum, C. | 2008 | Herpes simplex virus (HSV)-suppressive therapy decreases plasma and genital HIV-1 levels in HSV-2/HIV-1 coinfected women: a randomized, placebo-controlled, cross-over trial | South America | cross over RCT | 20 | Journal of Infectious Diseases | effect of valcyclovir on plasma and genital HIV (transmission) | valcyclovir was associated with reduced plasma and genital HIV. May be useful in reducing HIV transmission in dually infected |
| Anuradha, K.,Singh, H. M.,Gopal, K. V. T.,Rao, G. R. R.,Ramani, T. V.,Jyothi, Padmaja | 2008 | Herpes simplex virus 2 infection: a risk factor for HIV infection in heterosexuals | South Asia | case control study | 100 | Indian Journal of Dermatolog y, Venereology & Leprology | comparing prevalence of HSV in HIV pos and neg | prevalence of HSV significantly higher in HIV pos |

| | | | | | | | | |
|---|---|---|---|---|---|---|---|---|
| Glynn, J. R.,Crampin, A. C.,Ngwira, B. M. M.,Ndhlovu, R.,Mwanyongo, O.,Fine, P. E. M. | 2008 | Herpes simplex virus type 2 trends in relation to the HIV epidemic in northern Malawi | Southern Africa | cross sectional study | 999 | Sexually Transmitted Infections | long term trends of HSV-2 compared to HIV /natural history/prevalen ce | HSV2 was likely to already be highly prevalent before HIV epidemic. High HSV prevalence not likely to be a consequence of HIV |
| Benjamin, R. J.,Busch, M. P.,Fang, C. T.,Notari, E. P.,Puren, A.,Schoub, B. D.,Tobler, L. H.,Hogrefe, W.,Heyns, A. P. du,Stramer, S. L.,Crookes, R. L. | 2008 | Human immunodeficiency virus-1 infection correlates strongly with herpes simplex virus-2 (genital herpes) seropositivity in South African and United States blood donations | Southern Africa | cross sectional study | 625 | Transfusion | prevalence /epi | HSV 2 genital infection strongly correlated to HIV |

| | | | | | | | | |
|---|---|---|---|---|---|---|---|---|
| Bollen, L. J. M.,Whitehead, S. J.,Mock, P. A.,Leelawiwat, W.,Asavapiriyanont, S.,Chalermchockchareonkit, A.,Vanprapar, N.,Chotpitayasunondh, T.,McNicholl, J. M.,Tappero, J. W.,Shaffer, N.,Chuachoowong, R. | 2008 | Maternal herpes simplex virus type 2 coinfection increases the risk of perinatal HIV transmission: possibility to further decrease transmission? | East Asia | longitudinal survey | 307 | AIDS | effect of maternal genital herpes on perinatal HIV transmission/shedding in dually infected people | maternal HSV infection associated with increased risk of perinatal HIV transmission. Increased risk of intrapartum HIV transmission in those who were shedding. |
| Moira A. McMahon‡, Janet D. Siliciano§, Jun Lai¶, Jun O. Liu‡,James T. Stivers‡, Robert F. Siliciano§¶, and Rahul M. Kohli§ | 2008 | The Antiherpetic Drug Acyclovir Inhibits HIV Replication and Selects the V75I Reverse Transcriptase Multidrug Resistance Mutation* | sub saharan Africa | lab study | 999 | journal f biological chemistry | effect of acyclovir on ART resistance in HIV patients (V5721 gene) | episodic therapy with acyclovir may be associated with mutation in HIV reverse transcriptase genre leading to ART resistance. Clinicians may need to be cautious when starting dually infected people no t on haart on acyclovir |

| | | | | | | | | |
|---|---|---|---|---|---|---|---|---|
| Cowan, F. M.,Pascoe, S. J.,Barlow, K. L.,Langhaug, L. F.,Jaffar, S.,Hargrove, J. W.,Robinson, N. J.,Bassett, M. T.,Wilson, D.,Brown, D. W. G.,Hayes, R. J. | 2008 | A randomised placebo-controlled trial to explore the effect of suppressive therapy with acyclovir on genital shedding of HIV-1 and herpes simplex virus type 2 among Zimbabwean sex workers | Southern Africa | RCT | 125 | Sexually Transmitted Infections | effect of acyclovir on HIV and HSV genital shedding | acyclovir reduced HSV shedding but did not reduce HIV shedding. May be explained by low adherence or small sample size, larger clinical trials needed. |
| Nakubulwa, S.,Mirembe, F. M.,Kaye, D. K.,Kaddu-Mulindwa, D. | 2009 | Association between HSV-2 and HIV serostatus in pregnant women of known HIV serostatus attending Mulago Hospital antenatal clinic, Kampala, Uganda | East Africa | case control study | 250 | Journal of Infection in Developing Countries | compare HSV prevalence in HIV neg and pos pregnant women | odds of HSV positivity was almost 4 times higher in HIV pos (OR 3.7) |

| | | | | | | | | |
|---|---|---|---|---|---|---|---|---|
| Sobngwi-Tambekou, J.,Taljaard, D.,Lissouba, P.,Zarca, K.,Puren, A.,Lagarde, E.,Auvert, B. | 2009 | Effect of HSV-2 serostatus on acquisition of HIV by young men: results of a longitudinal study in orange farm, South Africa | Southern Africa | longutudinal study nested within a RCT | 2974 | Journal of Infectious Diseases | effect of HSV on HIV acquisition in men | prevalent HSV caused a increase in HIV acquisition (incident rate ratio = 3.3). Incident HSV had even greater effect (IRR= 7.0). Population attributable fraction of incident HIV caused by HSV was 27.8%. HSV does not alter the protective effect size of circumcision on HIV acquisition |

| | | | | | | | | |
|---|---|---|---|---|---|---|---|---|
| Gray, R. H.,Serwadda, D.,Tobian, A. A. R.,Chen, M. Z.,Makumbi, F.,Suntoke, T.,Kigozi, G.,Nalugoda, F.,Iga, B.,Quinn, T. C.,Moulton, L. H.,Laeyendecker, O.,Reynolds, S. J.,Kong, X. R.,Wawer, M. J. | 2009 | Effects of genital ulcer disease and herpes simplex virus type 2 on the efficacy of male circumcision for HIV prevention: analyses from the Rakai trials | East Africa | RCT | 2756 | PLoS Medicine | effect of HSV on the effectiveness of circumcision in preventing HIV acquisition | HSV di not have a significant effect on the effectiveness of circumcision to prevent HIV acquisition, suggesting that the protective effect mainly comes from removal of HIV target cells in the foreskin tissue. |

| | | | | | | | | |
|---|---|---|---|---|---|---|---|---|
| Johnson, K. E.,Sherman, M. E.,Ssempiija, V.,Tobian, A. A. R.,Zenilman, J. M.,Duggan, M. A.,Kigozi, G.,Serwadda, D.,Wawer, M. J.,Quinn, T. C.,Rabkin, C. S.,Gray, R. H. | 2009 | Foreskin inflammation is associated with HIV and herpes simplex virus type-2 infections in Rakai, Uganda | East Africa | cross sectional study nested within RCT | 233 | AIDS | effect of HSV andn HIV on foreskin inflammation | HSV and HIV increased foreskin inflammation separately and together to a greater extent. Foreskin inflammation may be implicated in HIV transmission and acquisition |
| Zuckerman, R. A.,Lucchetti, A.,Whittington, W. L. H.,Sánchez, J.,Coombs, R. W.,Magaret, A.,Wald, A.,Corey, L.,Celum, C. | 2009 | HSV suppression reduces seminal HIV-1 levels in HIV-1/HSV-2 co-infected men who have sex with men | South America | RCT | 20 | AIDS | effect of HSV therapy on seminal HIV shedding in dually infected men | valcyclovir therapy reduced seminal HIV shedding in dually infected msm in Peru. Another trial will be carried out to validate this with HIV transmission as an outcome |

| | | | | | | | | |
|---|---|---|---|---|---|---|---|---|
| Mayaud, P.,LeGoff, J.,Weiss, H. A.,Grésenguet, G.,Nzambi, K.,Bouhlal, H.,Frost, E.,Pépin, J.,Malkin, J. E.,Hayes, R. J.,Mabey, D. C. W.,Bélec, L. | 2009 | Clinical and virologic response to episodic acyclovir for genital ulAfrican women with herpes ulcers: a randomized placebo-controlled trial | West and central Africa | RCT | 441 | Journal of Infectious Diseases | effect of episodic acyclovir on HIV transmission parameters in dually infected | episodic acyclovir reduced cervicovaginal HSV shedding but did not reduce genital and plasma HIV rna loads. Trials of longer duration of therapy should be done |
| Freeman, Esther E.,White, Richard G.,Bakker, Roel,Orroth, Kate K.,Weiss, Helen A.,Buvé, Anne,Hayes, Richard J.,Glynn, Judith R. | 2009 | Population-level effect of potential HSV2 prophylactic vaccines on HIV incidence in sub-Saharan Africa | East West and Southern African cities | modelling study based on populatio n based data | 999 | Vaccine | effect of HSV vaccine on HIV incidence | HSV vaccine may have substantial effect on HIV incidence (theoretical). More should be done to make one available sooner |
| Baggaley, Rebecca F.,Griffin, Jamie T.,Chapman, Ruth,Hollingswort h, T. Déirdre,Nagot, Nicolas,Delany, | 2009 | Estimating the public health impact of the effect of herpes simplex virus suppressive therapy on plasma HIV-1 viral load | West and Southern Africa | modelling study | 999 | AIDS (London, England) | effect of HSV therapy on vira load | limited effect of HSV therapy on HIV viral load |

| | | | | | | | | |
|---|---|---|---|---|---|---|---|---|
| Sinead,Mayaud, Philippe,de Wolf, Frank,Fraser, Christophe,Ghani , Azra C.,Weiss, Helen A. | | | | | | | | |
| Baltzer, H.,Chege, D.,Rebbapragad a, A.,Wachihi, C.,Shin, L. Y. Y.,Kimani, J.,Ball, T. B.,Jaoko, W.,Plummer, F. A.,Kaul, R. | 2009 | Relative HIV resistance in Kenyan sex workers is not due to an altered prevalence or mucosal immune impact of herpes simplex virus type 2 infection | East Africa | cross sectional study | 139 | Current HIV Research | investigating whether HSV was implicated in relative HIV resistance in FSW in Kenya | relative HIV resistance was not likely to be due to decreased HSV prevalence or mucosal immune impact of genital HSV |
| Mahiane, S. G.,Legeai, C.,Taljaard, D.,Latouche, A.,Puren, A.,Peillon, A.,Bretagnolle, J.,Lissouba, P.,Nguéma, E. P. N.,Gassiat, E.,Auvert, B. | 2009 | Transmission probabilities of HIV and herpes simplex virus type 2, effect of male circumcision and interaction: a longitudinal study in a township of South Africa | Southern Africa | modelling study using longitudin al study data | 3274 | AIDS | the effect of HSV on female to male sexual transmission of HIV (and vice cerca). | HSV in either partner increased female to male risk of transmission of HIV (RR 3.0). HIV in either partner increased female to male risk of transmission of HSV (RR 2.5). |

| | | | | | | | | |
|---|---|---|---|---|---|---|---|---|
| Tobian AAR, Ssempijja V, Kigozi G, Oliver AE, Serwadda D, Makumbi F | 2009 | Incident HIV and herpes simplex virus type 2 infection among men in Rakai, Uganda. | Southern Africa | | | AIDS | | |
| Tanton, C.,Weiss, H. A.,Rusizoka, M.,LeGoff, J.,Changalucha, J.,Baisley, K.,Mugeye, K.,Everett, D.,Belec, L.,Clayton, T. C.,Ross, D. A.,Hayes, R. J.,Watson-Jones, D. | 2010 | Long-term impact of acyclovir suppressive therapy on genital and plasma HIV RNA in Tanzanian women: a randomized controlled trial | East Africa | RCT | 484 | Journal of Infectious Diseases | long term effect of acyclovir on HIV genital and plasma load in dually infected women | acyclovir did not show significant effect on HIV shedding parameters |

| | | | | | | | | |
|---|---|---|---|---|---|---|---|---|
| LeGoff, J.,Tanton, C.,Delaugerre, C.,Weiss, H. A.,Changalucha, J.,Ross, D. A.,Mugeye, K.,Belec, L.,Hayes, R. J.,Watson-Jones, D. | 2010 | No selection of nucleoside reverse transcriptase inhibitor resistance associated mutations by acyclovir suppressive therapy in herpes simplex virus-2/HIV-1 dually infected persons | East Africa | cross sectional study nested within RCT | 21 | | does acyclovir induce mutations that cause NRTI resistance in dually infected? | acyclovir did not cause the mutations in dually infected women from Tanzania who had received bd acyclovir 400mg for 12 months |
| C. Celum, A. Wald, J.R. Lingappa, A.S. Magaret, R.S. Wang, N. Mugo, A. Mujugira, J.M. Baeten, J.I. Mullins, J.P. Hughes, E.A. Bukusi, C.R. Cohen | 2010 | Acyclovir and Transmission of HIV-1 from Persons Infected with HIV-1 and HSV-2 | Southern Africa | RCT | 3408 | new England Journal of Medicine | effect of standard dose acyclovir on forward transmission of HIV in dually infected | although acyclovir reduced plasma HIV rna , it did not reduce HIV transmission in heterosexual HIV discordant couples |
| Munjoma, M. W.,Mapingure, M. P.,Stray-Pedersen, B. | 2010 | Risk factors for herpes simplex virus type 2 and its association with HIV among pregnant teenagers in Zimbabwe | Southern Africa | | 176 | Sexual Health | prevalence /epi | prevalence of HSV much higher in HIV infected (OR: 7.9) |

| | | | | | | | | |
|---|---|---|---|---|---|---|---|---|
| Vickerman, P.,Devine, A.,Foss, A. M.,Delany-Moretlwe, S.,Mayaud, P.,Meyer-Rath, G. | 2011 | The cost-effectiveness of herpes simplex virus-2 suppressive therapy with daily aciclovir for delaying HIV disease progression among HIV-1-infected women in South Africa | Southern Africa | modelling study using primary healthcare data, demographic data and participant data from a | 999 | Sexually Transmitted Diseases | cost effectivness (cost per lie year gained) of acyclovir daily in dually infected | acyclovir daily could be a cost effective strategy to extend women's lives who are dually infected and not started art. This is assuming acyclovir becomes cheaper and widely available at that price. |
| Charpentier, C.,Koyalta, D.,Ndinaromtan, M.,Tchobkréo, B.,Jenabian, M. A.,Day, N.,Si-Mohamed, A.,Weiss, H.,Bélec, L. | 2011 | Distribution of HIV-1 and HSV-2 epidemics in Chad revealing HSV-2 hot-spot in regions of high-risk HIV spread | central Africa | population based survey (national) | 548 | Journal of Infection in Developing Countries | national distribution of HIV and HSV in chad-prevalence | significant association between prevalence of HIV and HSV |

| | | | | | | | | |
|---|---|---|---|---|---|---|---|---|
| Baeten, J. M.,Lingappa, J.,Beck, I.,Frenkel, L. M.,Pepper, G.,Celum, C.,Wald, A.,Fife, K. H.,Were, E.,Mugo, N.,Sanchez, J.,Essex, M.,Makhema, J.,Kiarie, J.,Farquhar, C.,Corey, L. | 2011 | Herpes simplex virus type 2 suppressive therapy with acyclovir or valacyclovir does not select for specific HIV-1 resistance in HIV-1/HSV-2 dually infected persons | South America, Southern Africa, and East Africa | modelling study using RCT data (in vivo) | 168 | Journal of Infectious Diseases | does acyclovir or valcyclovir induce mutations that cause NRTI resistance in dually infected? (V751) | in vivo studies suggest that acyclovir and valcyclovir do NOT cause V751 mutations at normal dose (contrary to what mc mahon found in in vitro studies) |
| Mugwanya, K.,Baeten, J. M.,Mugo, N. R.,Irungu, E.,Ngure, K.,Celum, C. | 2011 | High-dose valacyclovir HSV-2 suppression results in greater reduction in plasma HIV-1 levels compared with standard dose acyclovir among HIV-1/HSV-2 coinfected persons: a randomized, crossover trial | East Africa | cross over RCT | 32 | Journal of Infectious Diseases | high dose valcyclovir vs standard dose acyclovir in reducing plasma HIV rna in dually infected | high dose valcyclovir reduced plasma HIV significantly more than standard dos acyclovir- high dose valcyclovir may be considered in the effort to delay clinical course of HIV and reduce HIV infectivity |

| | | | | | | | | |
|---|---|---|---|---|---|---|---|---|
| Foss, A. M.,Vickerman, P. T.,Mayaud, P.,Weiss, H. A.,Ramesh, B. M.,Reza-Paul, S.,Washington, R.,Blanchard, J.,Moses, S.,Lowndes, C. M.,Alary, M.,Watts, C. H. | 2011 | Modelling the interactions between herpes simplex virus type 2 and HIV: implications for the HIV epidemic in Southern India | South Asia (India) | | | Sexually Transmitted Infections | modelling HSV and HIV interaction: contribution of HSV-2 to HIV and modelled effect of a 'perfect' HSV suppressive treatment. PAF as a outcome | HSV-2 plays big role in transmission and acquisition of HIV in South India. Continuous suppressive HSV therapy may be useful in FSW and clients in South India. 35.5% of HIV incidence in FSW due to HSV-2. |
| Tanton, Clare,Weiss, Helen A.,LeGoff, Jerome,Changalucha, John,Clayton, Tim C.,Ross, David A.,Belec, Laurent,Hayes, Richard J.,Watson-Jones, Deborah | 2011 | Patterns of herpes simplex virus shedding over 1 month and the impact of acyclovir and HIV in HSV-2-seropositive women in Tanzania | East Africa | RCT | 78 | Sexually transmitted infections | effect of acyclovir on HIV shedding in dually infected | acyclovir did not significantly reduce HIV shedding in dually infected |

| | | | | | | | | |
|---|---|---|---|---|---|---|---|---|
| Camara, M.,Seydi, M.,Dieye, T. N.,Sow, P. S.,Mboup, S.,Kestens, L.,Jennes, W. | 2012 | Association between herpes simplex virus type 2 and HIV-1 in a population of married couples from Dakar, Senegal | West Africa | cross sectional study | 208 | International Journal of STD & AIDS | prevalence of HSV in different HIV couple status | strong association nin prevalence of HIV and HSV (OR 4.6) but no difference in HSV prevalence between HIV discordant and HIV concordant positive suggesting HSV is not a RF for HIV in this population |
| Ghebremichael, Musie,Habtzgi, Desale,Paintsil, Elijah | 2012 | Deciphering the epidemic synergy of herpes simplex virus type 2 (HSV-2) on human immunodeficiency virus type 1 (HIV-1) infection among women in sub-Saharan Africa | East arica | cross sectional study random sample | 1418 | BMC research notes | prevalence of HSV and HIV +association | HSV positive women more likely to be HIV positive (OR: 2.7) |
| Drake, A. L.; Roxby, A. C.; Ongecha-Owuor, F.; Kiarie, J.; John-Stewart, G.; Wald, A.; Richardson, B. A.; Hitti, J.; Overbaugh, J.; Emery, S.; Farquhar, C. | 2012 | Valacyclovir suppressive therapy reduces plasma and breast milk HIV-1 RNA levels during pregnancy and postpartum: a randomized trial | East Africa | RCT | 148 | Journal of Infectious Diseases | | |

| | | | | | | | | |
|---|---|---|---|---|---|---|---|---|
| Reynolds, S. J.,Makumbi, F.,Newell, K.,Kiwanuka, N.,Ssebbowa, P.,Mondo, G.,Boaz, I.,Wawer, M. J.,Gray, R. H.,Serwadda, D.,Quinn, T. C. | 2012 | Effect of daily aciclovir on HIV disease progression in individuals in Rakai, Uganda, co-infected with HIV-1 and herpes simplex virus type 2: a randomised, double-blind placebo-controlled trial | East Africa | RCT | 440 | Lancet Infectious Diseases | effect of acyclovir of hi disease progression (CD4 count as outcome) | acyclovir standard dose reduced rate of HIV disease progression in those with high baseline viral load |
| Roxby, A. C.,Drake, A. L.,Ongecha-Owuor, F.,Kiarie, J. N.,Richardson, B.,Matemo, D. N.,Overbaugh, J.,Emery, S.,John-Stewart, G. C.,Wald, A.,Farquhar, C. | 2012 | Effects of valacyclovir on markers of disease progression in postpartum women co-infected with HIV-1 and herpes simplex virus-2 | East Africa | RCT | 148 | PLoS ONE | effect of valcyclovir on HIV disease progression (CD4 and viral load as outcome measures) | valcyclovir was associated with greater increases in CD4 and smaller increases in viral load. Could be useful for pregnant women and delay need for ART |

| | | | | | | | | |
|---|---|---|---|---|---|---|---|---|
| Forhan, S. E.,Dunne, E. F.,Sternberg, M. R.,Whitehead, S. J.,Leelawiwat, W.,Thepamnuay, S.,Chen, C.,Evans-Strickfaden, T. T.,McNicholl, J. M.,Markowitz, L. E. | 2012 | HIV-1 and herpes simplex virus type-2 genital shedding among co-infected women using self-collected swabs in Chiang Rai, Thailand | East Asia | longitudinal survey | 67 | International Journal of STD & AIDS | self collected samples for HIV and HSV shedding in dually infected | SCS useful for future studies in dually infected since it allows more frequent sampling of more people. |
| Mawak JD, Dashe N, Atseye AB, Agabi YA, Zakeri H | 2012 | Seroprevalence and co-infection of herpes simplex virus type 2 and human immunodeficiency virus in Nigeria | West Africa | Cross sectional survey | | Shiraz E Medical Journal | | |

| | | | | | | | | |
|---|---|---|---|---|---|---|---|---|
| Janbakhash, A.,Mansouri, F.,Vaziri, S.,Sayad, B.,Afsharian, M.,Abedanpor, A. | 2012 | Seroepidemiology of herpes simplex virus type 2 (HSV2) in HIV infected patients in Kermanshah-Iran | South Asia | cross sectional study | 335 | Caspian Journal of Internal Medicine (CJIM) | prevalence | HSV prevalence in HIV positive people was low (6%) therefore HSV interventions arentnecessary for HIV management in this part of iran |
| Todd, J.,Riedner, G.,Maboko, L.,Hoelscher, M.,Weiss, H. A.,Lyamuya, E.,Mabey, D.,Rusizoka, M.,Belec, L.,Hayes, R. | 2013 | Effect of genital herpes on cervicovaginal HIV shedding in women co-infected with HIV and HSV-2 in Tanzania | East Africa | longitudinal survey | 131 | PLoS ONE | effect of HSV on HIV shedding in co infected | data suggests HSV is associated with increased HIV sheddig and hence transmissability (nor synonymous tho no). Data suggests that quantitiy o HSV genital shedding is a more prominent determinant of gential HIV shedding than presence of herpetic lesions. |
| Biraro, S.,Kamali, A.,White, R.,Karabarinde, A.,Ssendagala, J. N.,Grosskurth, H.,Weiss, H. A. | 2013 | Effect of HSV-2 on population-level trends in HIV incidence in Uganda between 1990 and 2007 | East Africa | retrospective cohort | 13,021 | Tropical Medicine and International Health | long term effect of population level HSV on population level HIV incidence: epi | high PAF (60% in males and 70% in females) in this pop. However no association found between trends of HSV and HIV but this may be due to HIV incidence not changing much across the time. |

| | | | | | | | | |
|---|---|---|---|---|---|---|---|---|
| Phiri, S.,Zadrozny, S.,Weiss, H. A.,Martinson, F.,Nyirenda, N.,Chen, ChengYen,Miller, W. C.,Cohen, M. S.,Mayaud, P.,Hoffman, I. F. | 2013 | Etiology of genital ulcer disease and association with HIV infection in Malawi | Southern Africa | cross sectional study | 422 | Sexually Transmitted Diseases | prevalence /epi | Hiv prevalence strongly associated with HSV . Hiv more prevalent in recurrent HIV rather than first episode |
| Muiru, Anthony N.,Guthrie, Brandon L.,Bosire, Rose,Merkel, Michele,Liu, Amy Y.,Choi, Robert Y.,Lohman-Payne, Barbara,Gatuguta, Ann,Mackelprang, Romel D.,Kiarie, James N.,Farquhar, Carey | 2013 | Incident HSV-2 infections are common among HIV-1-discordant couples | East Africa | prospective cohort study | 469 | The Journal of infectious diseases | effct of HIV on HSV incidence | highi prevalenc and incidence of HSV in HIV discordant couples in Kenya. Suggests that HSV therapy may be useful in reucing HSV and HIV transmission in high risk groups. |

| | | | | | | | | |
|---|---|---|---|---|---|---|---|---|
| Celum, C.,Morrow, R. A.,Donnell, D.,Hong, T.,Hendrix, C. W.,Thomas, K. K.,Fife, K. H.,Nakku-Joloba, E.,Mujugira, A.,Baeten, J. M. | 2014 | Daily oral tenofovir and emtricitabine-tenofovir preexposure prophylaxis reduces herpes simplex virus type 2 acquisition among heterosexual HIV-1-uninfected men and women: a subgroup analysis of a randomized trial | East Africa | sub analysis of a RCT | 1498 | Annals of Internal Medicine | effect of daily PrEP on HSV incidence | daily oral tenofovir based PrEP reduced rate of HSV acquisition in iheterosexaul men and women. This is an added benefit of tenofovir based prep on top of HIV prevention |
| Chamoun-Emanuelli, Ana M.,Bobardt, Michael,Moncla, Bernard,Mankowski, Marie K.,Ptak, Roger G.,Gallay, Philippe,Chen, Zhilei | 2014 | Evaluation of PD 404,182 as an anti-HIV and anti-herpes simplex virus microbicide | conducted in US with view of developing countries | in vitro study | 999 | Antimicrobial agents and chemotherapy | evaluation of new microbicide PD in treating HIV and HSV | was previously known to kill HIV but study found that is it also effective in destroying HSV. Other tests suggest it is suitable for further investigation to becoming licensed virucidal |

| | | | | | | | | |
|---|---|---|---|---|---|---|---|---|
| Kenyon C, Buyze J, Colebunders R. | 2014 | Classification of incidence and prevalence of certain sexually transmitted infections by world regions | Multi | | | International journal of infectious diseases | prevalence /epi | |
| Kenyon, C. R.,Osbak, K.,Buyze, J. | 2014 | The prevalence of HIV by ethnic group is correlated with HSV-2 and syphilis prevalence in Kenya, South Africa, the United Kingdom, and the United States | East and Southern Africa | cross sectional study | >20,000 | Interdisciplinary Perspectives on Infectious Diseases | prevalence /epi | prev of HSV more common in HIV positive |
| Meque, I.,Dubé, K.,Feldblum, P. J.,Clements, A. C. A.,Zango, A.,Cumbe, F.,Chen, P. L.,Ferro, J. J.,Wijgert, J. H. van de | 2014 | Prevalence, incidence and determinants of herpes simplex virus type 2 infection among HIV-seronegative women at high-risk of HIV infection: a prospective study in beira, Mozambique | Southern Africa | prospective cohort study | 411 | PLoS ONE | risk of HIV in HSV pts | no association between beingHSV positive at baseline and acquiring HIV |

| | | | | | | | | |
|---|---|---|---|---|---|---|---|---|
| Ankamma, A.,Nagaraja, B.,Radha, P.,Reddy, B. K.,Venkatarao, B. | 2014 | Study of seroprevalence of HSV-2 among HIV seropositive individuals at S.V.R.R.G.G.H Tirupati | South Asia | cross sectional study | 182 | International Journal of Pharmaceutical Research and Bio-Science | prev | high prevalence in caes compared to control (sig) |
| Nakku-Joloba E, Kambugu F, Wasubire J, Kimeze J, Salata R, Albert JM | 2014 | Sero-prevalence of herpes simplex type 2 virus (HSV-2) and HIV infection in Kampala, Uganda | East Africa | cross sectional study | | African Health Sciences | prev | |
| Behling, Juliane,Chan, Adrienne K.,Zeh, Clement,Nekesa, Carolyne,Heinzerling, Lucie | 2015 | Evaluating HIV Prevention Programs: Herpes Simplex Virus Type 2 Antibodies as Biomarker for Sexual Risk Behavior in Young Adults in Resource-Poor Countries | East Africa | cross sectional study | 70,000 | PloS one | the use of HSV incidence as a proxy marker for sexual risk behaviour and HIV infection (because HIV incidence is much lower and therefore a huge sample size would be required) | the anticipated association between HSV serostatus and sexual risk behaviour was not found. Likely due to social bias from the interviews on sexual behaviour. Still some potential though for HSV serostatus as a marker of HIV |

| | | | | | | | | |
|---|---|---|---|---|---|---|---|---|
| Saramma, Jacob,Thatchinamoorthy, Gopal,Sivasangeetha, Kanagasabai,Anitha, Durairaj,Kamala, Sushi,Ganesan, Arumugam | 2015 | Herpes Simplex Virus 2 infection in HIV-seropositive individuals in Tamil Nadu, India | South Asia | longitudinal survey | 273 | International Journal of Medical Science and Public Health | prevalence of HSV in HIV pos pts | HSV significantly more prevalent in HIV positive . More so in women than men. |
| Okango, E.,Mwambi, H.,Ngesa, O.,Achia, T. | 2015 | Semi-parametric spatial joint modeling of HIV and HSV-2 among women in Kenya | East Africa | modelling study | 999 | PLoS ONE | joint spatial modelling the relationship between HIV and HSV | positive significant spatial relationship between HIV and HSV |
| Bharathi, V. C.,Anaparty, Usharani | 2015 | Sero evaluation of coinfection of HIV and HSV-2 | South Asia | cross sectional study | 130 | Journal of Pharmaceutical and Scientific Innovation (JPSI) | prevalence of HSV in HIV pos pts | high HSV prevalence in HIV positive (no control group though) |

| | | | | | | | | |
|---|---|---|---|---|---|---|---|---|
| Low, A. J.,Nagot, N.,Weiss, H. A.,Konate, I.,Kania, D.,Segondy, M.,Meda, N.,Perre, P. van de,Mayauda, P. | 2016 | Herpes simplex virus type-2 cervicovaginal shedding among women living with HIV-1 and receiving antiretroviral therapy in Burkina Faso: an 8-year longitudinal study | West Africa | cohort study | 236 | Journal of Infectious Diseases | effect of ART on vaginal shedding of HSV in dually infected | ART was associated with reduced genital HSV shedding over the 8 years. |
| Thienkrua, W.,Todd, C. S.,Chonwattana, W.,Wimonsate, W.,Chaikummao, S.,Varangrat, A.,Chitwarakorn, A.,Griensven, F. van,Holtz, T. H. | 2016 | Incidence of and temporal relationships between HIV, herpes simplex II virus, and syphilis among men who have sex with men in Bangkok, Thailand: an observational cohort | East Asia | cohort study | 960 | BMC Infectious Diseases | temporal relationship between HIV and HSV | high incidence of HIV and HSV in this cohort. Timeframe (60m) was not enough to assess the temporal relationships between HSV and HIV |
| Daniels, B.,Wand, H.,Ramjee, G. | 2016 | Prevalence of Herpes Simplex Virus 2 (HSV-2) infection and associated risk factors in a cohort of HIV negative women in Durban, South Africa | Southern Africa | cohort study withi a RCT | 2236 | BMC Research Notes | prevalence of HSV and HIV incidence | very high baseline HSV prevalence (65%). Hiv incidence significantly higher in those with prevalent HSV |

| | | | | | | | | |
|---|---|---|---|---|---|---|---|---|
| Nwadike, V. U.,Anaedobe, C. G.,Azeez, R. A.,Jinadu, S. S.,Chigozie, J. O. | 2018 | Atypical presentation of genital herpes in a retroviral disease patient on highly active anti-retroviral therapy | West Africa | case study | 1 | African Journal of Clinical and Experimenta l Microbiology | is haart associated with increased risk of acyclovir resistant genital herpes and chrionic genita herpes? | HIV positive people on haart may have more severe forms of HSV and clinicalns should consider offering acyclovir |
| Kenyon, C. R. | 2018 | HIV prevalence by ethnic group covaries with prevalence of herpes simplex virus-2 and high-risk sex in Uganda: an ecological study | East Africa | cross sectional study populatio n based | 11454 | PLoS ONE | association between HIV prevalence and HSV prevalece | highlyl correlated |
| Adejumo, B. I. G.,Oronsaye, F. E.,Drisu, U. I.,Adebowale, M. O.,Oke, O. M.,Dimkpa, U.,Omosor, K. I.,Abdulrahman, O. N.,Ukatu, E. N.,Michael, E. A. | 2018 | The level of CD4+ T cell count among reproductive age women coinfected with human immune virus, hepatitis surface antigen and herpes simplex virus in Kogi state, Nigeria | West Africa | cross sectional study | 342 | Health | is CD4 count affected by HSV and HIV coinfection | no evidence for this |

| | | | | | | | | |
|---|---|---|---|---|---|---|---|---|
| Mohraz, M.,Aghakhani, A.,Moayedi-Nia, S.,Banifazl, M.,Janbakhsh, A.,Mamishi, S.,Karami, A.,Bavand, A.,Mirzapour, P.,Ramezani, A. | 2018 | No role of herpes simplex virus type 2 (HSV-2) infection on HIV progression in naïve HIV patients | South Asia | cohort study | 201 | Iranian Biomedical Journal | effect of HSV in HIV disease progression | no evidence that hcoinfection accelerates HIV progression |
| Lima, L. R. P.,Almeida, N. A. A. de,Guterres, A.,Pilotto, J. H.,Niel, C.,Paula, V. S. de | 2018 | Novel variants of human herpesvirus 2 from Brazilian HIV-1 coinfected subjects | South America | cross sectional study | 6 | Memórias do Instituto Oswaldo Cruz | new variants of HSv-2 | new variant- should be further investigated |

| | | | | | | | | |
|---|---|---|---|---|---|---|---|---|
| Bradley, J.,Floyd, S.,Piwowar-Manning, E.,Laeyendecker, O.,Young, A.,Bell-Mandla, N.,Bwalya, J.,Bock, P.,Fidler, S.,Ayles, H.,Hayes, R. J. | 2018 | Sexually transmitted bedfellows: exquisite association between HIV and herpes simplex virus type 2 in 21 communities in Southern Africa in the HIV Prevention Trials Network 071 (PopART) study | Southern Africa | RCT | 38691 | Journal of Infectious Diseases | effect of HSV-2 on HIV inciedence | HIV incidence much more common in HSV-2 positive people (Or 6.6). Strong association between the prevalence of both on an individual and community level. Hsv interventions should be incorporated into HIV prevention |
| Madebe, R.,Kiwelu, I.,Ndaro, A.,Francis, F.,Baraka, V.,Theilgaard, Z.,Katzenstein, T. | 2020 | Herpes Simplex virus type 2 seroprevalence and risk factors among adolescents and youth with HIV-1 in Northern, Tanzania | East Africa | cross sectional study | 180 | Journal of Infection in Developing Countries | prevalence of HSV in HIV pos pts | oveall prevalence of HSV amongst the HIV pos youths was 18% but varried a lot by age- those between 20-14 had a six fold higher odds of being HSV pos than for 10-14 yr olds . Age specific interventions are important |

Mathematical modelling

| **Authors** | **Year** | **Title** | **Geographical area of study participants** | **Study topic** | **Main findings** |
|---|---|---|---|---|---|
| Korenromp, Eline L.,Bakker, Roel,De Vlas, Sake J.,Robinson, N. Jamie,Hayes, Richard,Habbema, J. Dik F. | 2002 | Can behaviour change explain increases in the proportion of genital ulcers attributable to herpes in sub-Saharan Africa? A simulation modeling study | East Africa (Tanzania) | proportion of GUd attributable to HSV: the effect of HIV and behaviour change on HSV incidence | increased proportion of GUD attributable to HSV likely to be artefact of increased detection etc not absolute (not caused by hv epidemic). Behavioural change in response to HIV epidemic (more condom use, less transactional sex) may have significant effect on increasing relative HSV cases (less of other treatable STIs/better treatment) |
| Korenromp, E. L.,Bakker, R.,Vlas, S. J. de,Gray, R. H.,Wawer, M. J.,Serwadda, D.,Sewankambo, N. K.,Habbema, J. D. F. | 2002 | HIV dynamics and behaviour change as determinants of the impact of sexually transmitted disease treatment on HIV transmission in the context of the Rakai trial | East Africa (India) | does HIV dynamics and behaviour change affect the impact of HSV treatment on HIV transmission | impact of HSV treatment on HIV transmission decreases with time in the first decade of an epidemic. in the second decade of a simulated epidemic the behaviour change may be a greater determinant of the impact of HSV therapy on HIV transmission than the stage of the epidemic. Timing and behaviour change are important things to consider before strating a HSV treatment for HIV transmission. |
| Freeman, E. E.,Orroth, K. K.,White, R. G.,Glynn, J. R.,Bakker, R.,Boily, M. C.,Habbema, D.,Buvé, A.,Hayes, R. | 2007 | Proportion of new HIV infections attributable to herpes simplex 2 increases over time: simulations of the changing role of sexually transmitted infections in sub- | East Africa, West Africa, Southern Africa (SSA) | impact of HSV on HIV incidence- population attributable fraction (PAF) as a outcome | PAF of HIV caused by HSV incresed with time in the modelled epidemic. Hsv has only small impact on nHIV incidence in early epidemic but a larger impact as the epidemic deelops and in core groups. Hsv interventions may be important later in epidemic of HIV. |

| | | | | | |
|---|---|---|---|---|---|
| | | Saharan African HIV epidemics | | | |
| Abu-Raddad, L. J.,Magaret, A. S.,Celum, C.,Wald, A.,Longini, I. M., Jr.,Self, S. G.,Corey, L. | 2008 | Genital herpes has played a more important role than any other sexually transmitted infection in driving HIV prevalence in Africa | East Africa, West Africa, Southern Africa (SSA) | role of HSV in driving HIV epidemic | HSV played a bigger role than any other STI in driving the Africa HIV epidemic. PAF (population attributable fraction) of HIV caused by HSV estimated to be over 25% in Kisumu Kenya. Support need for an HSV vaccine |
| Vickerman, P.,Ndowa, F.,Mayaud, P. | 2008 | Modelling the cost per ulcer treated of incorporating episodic treatment for HSV-2 into the syndromic algorithm for genital ulcer disease | sub saharan Africa input | cost effectiveness of incorporating acyclovir into syndromic management of GUD | incorporating acyclovir into syndromic management may be an affordable way of managing HSV correctly in low resource settings,. Prevalence of HSV-2 in the area may help determine whether or not to do this. |
| White, R. G.,Freeman, E. E.,Orroth, K. K.,Bakker, R.,Weiss, H. A.,O'Farrell, N.,Buvé, A.,Hayes, R. J.,Glynn, J. R. | 2008 | Population-level effect of HSV-2 therapy on the incidence of HIV in sub-Saharan Africa | West and East Africa | effect of HSV therapeutics on HIV incidence (acquisition and transmission)- epi | HSV therapy could have a positive impact on HIV incidence on population level in more concentrated epidemics, but only with high coverage long duration of therapy. Ideally a vaccine is needed. PAF of HSV on HIV is higher in more concentrated epidemics. |
| Foss, A. M.,Vickerman, P. T.,Chalabi, Z.,Mayaud, P.,Alary, M.,Watts, C. H. | 2009 | Dynamic modeling of Herpes simplex virus type-2 (HSV-2) transmission: issues in structural uncertainty | South Asia (India) | transmission modelling: structural issues | simple model is as accurate as complex model in developed HSV epidemics (>60% prev) |

| | | | | | |
|---|---|---|---|---|---|
| Freeman, E. E.,White, R. G.,Bakker, R.,Orroth, K. K.,Weiss, H. A.,Buvé, A.,Hayes, R. J.,Glyn, J. R. | 2009 | Population-level effect of potential HSV2 prophylactic vaccines on HIV incidence in sub-Saharan Africa | East West and Southern African cities | effect of HSV vaccine on HIV incidence | HSV vaccine may have substantial effect on HIV incidence (theoretial). More should be done to make one available sooner |
| Mahiane, S. G.,Legeai, C.,Taljaard, D.,Latouche, A.,Puren, A.,Peillon, A.,Bretagnolle, J.,Lissouba, P.,Nguéma, E. P. N.,Gassiat, E.,Auvert, B. | 2009 | Transmission probabilities of HIV and herpes simplex virus type 2, effect of male circumcision and interaction: a longitudinal study in a township of South Africa | South Africa | what is the rate of female to male sexual transmission of HSV and how is it affected by male circumcision? | HSV 2 increased risak of female to male sexual transmission of HIV. HIV increased risk of sexual transmission of HSV-2. MC reduced risk of HSV-2 transmission. Estimated tansmission rate of HSV-2 : 0.0067 in Africa |
| Alsallaq, R. A.,Schiffer, J. T.,Longini, I. M., Jr.,Wald, A.,Corey, L.,Abu-Raddad, L. J. | 2010 | Population level impact of an imperfect prophylactic vaccine for Herpes simplex virus-2 | developing country | population and individual effects of HSV-2 vaccine | pre exposure HSV 2 vaccine would only have a small effect on HSV 2 incidence. They would hav emuch greater public health benefits in developing countries where prevalence is higher. |
| Vickerman, P.,Devine, A.,Foss, A. M.,Delany-Moretlwe, S.,Mayaud, P.,Meyer-Rath, G. | 2011 | The cost-effectiveness of herpes simplex virus-2 suppressive therapy with daily aciclovir for delaying HIV disease progression among HIV-1-infected | Southern Africa | cost effectiveness of HSV suppressive therapy on HIV progression in co infected | HSV suppressive therapy could be a cost effective way to reduce HIV progression before ART initiation , this is based on cheap acyclovir being available |

| | | | | | |
|---|---|---|---|---|---|
| | | women in South Africa | | | |
| Foss, A. M.,Vickerman, P. T.,Mayaud, P.,Weiss, H. A.,Ramesh, B. M.,Reza-Paul, S.,Washington, R.,Blanchard, J.,Moses, S.,Lowndes, C. M.,Alaryj, M.,Watts, C. H. | 2011 | Modelling the interactions between herpes simplex virus type 2 and HIV: implications for the HIV epidemic in Southern India | South Asia (India) | modelling HSV and HIV interaction: contribution of HSV-2 to HIV and modelled effect of a 'perfect' HSV suppressive treatment. PAF as a outcome | HSV-2 plays big role in transmission and acquisition of HIV in South India. Continuous suppressive HSV therapy may be useful in FSW and clients in South India. 35.5% of HIV incidence in FSW due to HSV-2. |
| Arora P, Nagelkerke N, Sgaier SK, Kumar R, Dhingra N, Jha P | 2011 | HIV, HSV-2 and syphilis among married couples in India: patterns of discordance and concordance | South Asia | Modelling concordance and discordance in couples in India | |
| Kacker, S.,Frick, K. D.,Quinn, T. C.,Gray, R. H.,Tobian, A. A. R. | 2013 | Financial implications of male circumcision scale-up for the prevention of HIV and other sexually transmitted infections in a sub- | East Africa (India) | effect of MC on STIs including HSV-2 | STIs other than HIV (including HSV-2) also contribute to cost savings associated with increased MC rate. |

| | | | | | |
|---|---|---|---|---|---|
| | | Saharan African community | | | |
| Todd J, Riedner G, Maboko L, Hoelscher M, Weiss HA, Lyamuya E | 2013 | Effect of genital herpes on cervicovaginal HIV shedding in women co-infected with HIV and HSV-2 in Tanzania | East Africa | effect of HSV on HIV shedding in co infected | data suggests HSV is associated with increased HIV sheddig and hence transmissability (nor synonymous tho no). Data suggests that quantitiy o HSV genital shedding is a more prominent determinant of gential HIV shedding than presence of herpetic lesions. |
| Terris-Prestholt F, Foss AM, Cox AP, Heise L, Meyer-Rath G, Delany-Moretlwe S | 2014 | Cost-effectiveness of tenofovir gel in urban South Africa: model projections of HIV impact and threshold product prices | Southern Africa | Cost effectiveness models | |
| Jewell, B. L.,Cremin, I.,Pickles, M.,Celum, C.,Baeten, J. M.,Delany-Moretlwe, S.,Hallett, T. B. | 2015 | Estimating the cost-effectiveness of pre-exposure prophylaxis to reduce HIV-1 and HSV-2 incidence in HIV-serodiscordant couples in South Africa | South Africa | cost effectiveness of daily tenofovir PrEP for reducing HSV2 incidence | the protective effects of PrEP against HSV-2 does not significantly improve the cost effectiveness of PrEP. But the modest improvement may be an additional factor that makes this more appealing as a HIV intervention. |

| | | | | | |
|---|---|---|---|---|---|
| Ghebremichael, M. | 2015 | Joint modelling of correlated binary outcomes: HIV-1 and HSV-2 co-infection | East Africa (Tanzania) | HIV and HSv 2 coinfection usinf joint response model that accounts for the inter-dependence of both infections | joint response model is more accurate ans less biased than modeling both diseases separately |
| Okango, E.,Mwambi, H.,Ngesa, O.,Achia, T. | 2015 | Semi-parametric spatial joint modeling of HIV and HSV-2 among women in Kenya | East Africa | joint spatial modelling the relationship between HIV and HSV | positive significant spatial relationship between HIV and HSV |
| Okango, E.,Mwambi, H.,Ngesa, O. | 2016 | Spatial modeling of HIV and HSV-2 among women in Kenya with spatially varying coefficients | East Africa | relaxing assumptions in spatial modelling of HSV | spatial variation in determinants of HSV occurred. using models to relax assumptions can result in more accurate results and interpretations. |
| Omori, R.,Abu-Raddad, L. J. | 2017 | Sexual network drivers of HIV and herpes simplex virus type 2 transmission | East Africa (Kenya, India) and many SSA countries | effect of key sexual network characteristics on HSV transmission. Is HSV prevalence a good proxy for HIV prevalence. | HSV-2 prevalence could be used as HIV prevalence proxy. Many sexual network predictors of HSV |
| Abbai, Nathlee S.,Nyirenda, Makandwe,Naidoo, Sarita,Ramjee, Gita | 2018 | Prevalent Herpes Simplex Virus-2 Increases the Risk of Incident Bacterial Vaginosis in Women from South Africa | Southern Africa | effect of prevalent HSV on BV incidence | HSV positive women at higher risk of developing BV (AOR: 1,17) |
| Stoner MCD, Neilands TB, Kahn K, Hughes JP, | 2019 | Multilevel measures of education and pathways to incident | Southern Africa | | |

| | | | | | |
|---|---|---|---|---|---|
| Gómez-Olivé FX, Twine R | | Herpes simplex virus type 2 in adolescent girls and young women in South Africa | | | |
| Looker, K. J.,Johnston, C.,Welton, N. J.,James, C.,Vickerman, P.,Turner, K. M. E.,Boi, M. C.,Gottlieb, S. L. | 2020 | The global and regional burden of genital ulcer disease due to herpes simplex virus: a natural history modelling study | global including Africa, Asia, Americas | proportion of GUD attributable to HSV | estimated 178 million people aged 15-49 experienced 1 or more GUD caused by HSV-2 in the world in 2016 (almost 5% of world pop). Highest in Africa and double in women than men. |
