# Supplementary material for: 20 years of herpes simplex virus type 2 (HSV-2) research in low-income and middle-income countries: systematic evaluation of progress made in addressing WHO priorities for research in HSV-2/HIV interactions, HSV-2 control and mathematical modelling
Source: BMJ Glob Health. 2024 Jul 4;9(7):e015167. doi: 10.1136/bmjgh-2024-015167 (PMC11227757; doi:10.1136/bmjgh-2024-015167)
Supplement: Supplementary data [file bmjgh-2024-015167supp003.pdf]

Appendix 3 - Reasons for exclusion after full-text eligibility review for each research area

Control measures

Number of excluded records: 30

Reasons for exclusions: Studies on HIV prevention without recording of effect on HSV-2 (n=6), STI prevention without HSV-2 data (n=4), developed countries (n=10), studies on HSV-1 prevention (n=10)

HSV-2/HIV interaction

Number of excluded records: 4

Reasons for exclusions: 4 discussed HSV-2 and HIV independently (not the interactions)

Modelling

Number of excluded records: 21

Reasons for exclusions: 21 were not mathematical modelling studies- they simply used statistical modelling to analyse their own data
